# *Symbiodinium* genomes reveal adaptive evolution of functions related to coral-dinoflagellate symbiosis

**DOI:** 10.1038/s42003-018-0098-3

**Published:** 2018-07-17

**Authors:** Huanle Liu, Timothy G. Stephens, Raúl A. González-Pech, Victor H. Beltran, Bruno Lapeyre, Pim Bongaerts, Ira Cooke, Manuel Aranda, David G. Bourne, Sylvain Forêt, David J. Miller, Madeleine J. H. van Oppen, Christian R. Voolstra, Mark A. Ragan, Cheong Xin Chan

**Affiliations:** 1 Institute for Molecular Bioscience, The University of Queensland, Brisbane, QLD 4072 Australia; 2 Australian Institute of Marine Science, Townsville, QLD 4810 Australia; 3 ARC Centre of Excellence for Coral Reef Studies, James Cook University, Townsville, QLD 4811 Australia; 4 Department of Molecular and Cell Biology, James Cook University, Townsville, QLD 4811 Australia; 5 Global Change Institute, The University of Queensland, Brisbane, QLD 4072 Australia; 6 Institute for Biodiversity Science and Sustainability, California Academy of Sciences, San Francisco, CA 94118 USA; 7 Red Sea Research Center, Division of Biological and Environmental Science and Engineering, King Abdullah University of Science and Technology (KAUST), Thuwal, 23955-6900 Saudi Arabia; 8 College of Science and Engineering, James Cook University, Townsville, QLD 4811 Australia; 9 Research School of Biology, Australian National University, Canberra, ACT 2601 Australia; 10 School of BioSciences, The University of Melbourne, VIC, 3010 Australia; 11 School of Chemistry and Molecular Biosciences, The University of Queensland, Brisbane, QLD 4072 Australia; 12 Present Address: Laboratoire d’excellence CORAIL, Centre de Recherches Insulaires et Observatoire de l’Environnement, Moorea, 98729 French Polynesia

## Abstract

Symbiosis between dinoflagellates of the genus *Symbiodinium* and reef-building corals forms the trophic foundation of the world’s coral reef ecosystems. Here we present the first draft genome of *Symbiodinium goreaui* (Clade C, type C1: 1.03 Gbp), one of the most ubiquitous endosymbionts associated with corals, and an improved draft genome of *Symbiodinium kawagutii* (Clade F, strain CS-156: 1.05 Gbp) to further elucidate genomic signatures of this symbiosis. Comparative analysis of four available *Symbiodinium* genomes against other dinoflagellate genomes led to the identification of 2460 nuclear gene families (containing 5% of *Symbiodinium* genes) that show evidence of positive selection, including genes involved in photosynthesis, transmembrane ion transport, synthesis and modification of amino acids and glycoproteins, and stress response. Further, we identify extensive sets of genes for meiosis and response to light stress. These draft genomes provide a foundational resource for advancing our understanding of *Symbiodinium* biology and the coral-algal symbiosis.

## Abstract (web summary)

Huanle Liu et al. report draft genomes of two *Symbiodinium* species, one from the most dominant type of symbionts in reef-building corals. They find evidence of positive selection in genes related to stress response, meiosis and other traits required for forming successful symbiotic relationships.

## Introduction

Coral reefs provide habitats for one-quarter to one-third of all marine species^1^. Although typically surrounded by nutrient-poor waters, coral reefs show high rates of primary productivity, with the fixed carbon supporting not only the biomass of reef organisms but also commercial and recreational fisheries. Reef-building corals rely on the symbiosis between the coral animal per se and photosynthetic dinoflagellates of the genus *Symbiodinium*. This symbiosis is based on mutual nutrient exploitation, with corals providing shelter and inorganic nutrients to their algal partners, while *Symbiodinium* supply their coral hosts with photosynthates that can meet up to 95% of the corals’ energy requirements^2^.

The relationship between *Symbiodinium* and their host determines not only the rate of coral-reef growth (calcium carbonate deposition), but also how the system responds to environmental stress^2^. Many studies have shown that coral-*Symbiodinium* mutualism is susceptible to environmental factors including temperature, light and salinity^3^. Exposure to ultraviolet radiation, thermal stress or a combination of both can initiate photoinhibition, decoupling of carbon flow between symbiont and host, oxidative damage and breakdown of the symbiosis, a phenomenon known as coral bleaching. Unless the symbiosis is soon re-established the coral host is at risk of starvation, disease and eventual death. In recent decades, coral bleaching has led to large-scale mortality on coral reefs around the world, with the most recent global coral bleaching event (2014–2017) now confirmed as the longest and most severe on record^4^.

Despite the critical importance of this coral-dinoflagellate symbiosis, little is known about the underlying molecular mechanisms (apart from photosynthesis and carbon exchange), largely due to the lack of comprehensive understanding of what molecules, pathways and functions *Symbiodinium* can contribute. Genomes of dinoflagellates are known for their idiosyncratic features including non-canonical splice sites, extensive methylation^5^ and large sizes, up to 250 Gbp^6^. Their plastid genomes occur as plasmid-like minicircles^7^; their mitochondrial genomes harbour only three protein-coding genes and lack stop codons^8^, and both mitochondrial and nuclear^9^ transcripts are extensively edited.

*Symbiodinium* are classified into nine clades^10^, with members of Clades A, B, C and D responsible for the vast majority of associations with scleractinian corals^11^. Draft genomes have been published for representatives of Clades A, B, C and F^12–15^, with sequence comparisons demonstrating *Symbiodinium* isolates (and clades) to be highly divergent^13,16^. With the exception of a recently published draft genome of the foraminifera-associated *Symbiodinium* sp. Y103^15^, genome sequences are still lacking for Clade C, the most ubiquitous and diverse clade associated with tropical reef corals^17^, at least some sub-clades (types) of which are ecologically partitioned^18^.

Here we report draft genomes of two *Symbiodinium* from the Pacific Ocean: *Symbiodinium goreaui* (type C1; isolated from the acroporid coral *Acropora tenuis*) from the Great Barrier Reef, and *Symbiodinium kawagutii* CS-156 (=CCMP2468, Clade F) from Hawaii. *Symbiodinium* type C1 is one of two living ancestors (along with type C3) of Clade C^17^, and one of the most dominant type associated with reef corals in both Indo-Pacific and Caribbean waters. *S. goreaui* has been reported from >150 coral species on Australia’s Great Barrier Reef, representing >80% of the studied coral genera in this region across environments from reef flats to lower mesophotic depths^19,20^. In contrast, *S. kawagutii* CS-156 (=CCMP2468) was isolated during attempts to culture the symbiont from *Montipora verrucosa* (Todd LaJeunesse, *personal communication*). This isolate has yet to be verified to occur in mutualistic symbiosis with any coral, and appears incapable of establishing experimental symbiosis with cnidarian hosts^21^. Instead, *S. kawagutii* may be exclusively a symbiont of foraminifera, or occur free-living at low environmental densities, but proliferate opportunistically in culture. As some genome data have been published for *S. kawagutii* CCMP2468^13^, we used these in combination with new data from the present study to generate a refined genome assembly. The genomes of *S. goreaui* and *S. kawagutii* offer a platform for comparative genomic analyses between two of the most-recently diverged *Symbiodinium* lineages Clades C and F, and published genome sequences in the more-basal Clades A and B.

Adopting a comparative approach using both genome and transcriptome data, we systematically investigated genes and functions that are specific to *Symbiodinium* in relation to other dinoflagellates, and their association with the establishment and maintenance of symbiosis. We computationally identify genes and functions for which there is evidence of adaptive selection in *Symbiodinium*. We also identify extensive sets of genes for meiosis and response to light stress. Our results indicate adaptive selection in *Symbiodinium* gene functions that are related to establishment of cnidarian-dinoflagellate symbiosis, and provide compelling genomic evidence (based on gene repertoire) that *Symbiodinium* is, or has recently been, capable of meiosis. To our knowledge, this is the most comprehensive comparative analysis so far of *Symbiodinium* genomes, and the first to include a prominent endosymbiont of corals of Indo-Pacific and Caribbean reefs.

## Results

### Genomes of *S. goreaui* and *S. kawagutii*

We sequenced and generated two draft *Symbiodinium* genome assemblies de novo, for *S. goreaui* (Clade C, 1.03 Gbp) and for *S. kawagutii* (Clade F, 1.05 Gbp). Details of data generation and assembly statistics are shown in Supplementary Tables 1 and 2, respectively. Our *S. goreaui* assembly consists of 41,289 scaffolds (N50 length 98,034 bp). For *S. kawagutii*, we first verified that our data (from isolate CS-156) and the published data (from the synonym isolate CCMP2468) are indeed from the same culture of origin (see Methods and Supplementary Fig. 1). Compared to the published assembly by Lin et al.^13^, independent mapping of their ten fosmid sequences^13^ onto our preliminary CS-156 assembly yielded up to 43-fold and 37-fold fewer gaps and mismatches, respectively (Supplementary Fig. 2). We later combined both datasets in a single de novo assembly, yielding 16,959 scaffolds (N50 length 268,823 bp). Genome-size estimates based on *k*-mer coverage are 1.19 Gbp for *S. goreaui* and 1.07 Gbp for *S. kawagutii* (Supplementary Table 3), comparable to those for other sequenced *Symbiodinium* genomes. We also recovered sequences putatively derived from their plastid genomes (Supplementary Tables 4, 5 and 6), including their distinct core conserved regions (Supplementary Table 7), and from their mitochondrial genomes; see Supplementary Note 1 for details.

The repeat content of the assembled genomes ranged from 16.0% (*S. kawagutii*) to 27.9% (*Symbiodinium microadriaticum*); a large peak in transposable element (TE) abundance observed at high divergence (Kimura distance^22^ 15–25) in all genomes (Supplementary Fig. 3) suggests that most extant TEs are remnants of an ancient burst of TE activity that had occurred before the diversification of *Symbiodinium*. In all genomes, the proportion of long interspersed nuclear elements is larger than that of long terminal repeats. TE activity has been broadly linked to genome size in plants^23^, so reduced TE activity may be connected with the relative compactness of *Symbiodinium* genomes in comparison to those of other dinoflagellates. However, as these genomes are still in draft, the impact of assembly completeness on the patterns of repeat divergence cannot be dismissed.

**Fig. 1:**
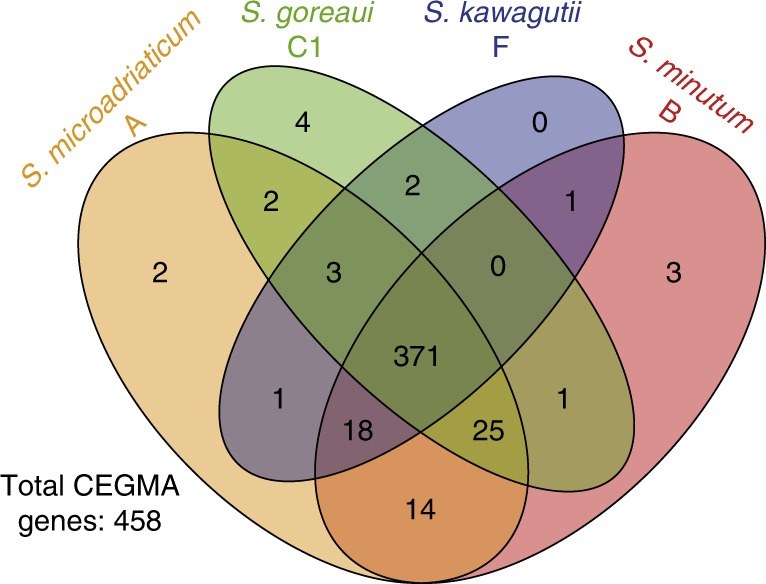
Comparison of *Symbiodinium* genomes. Number of recovered core eukaryote genes in each genome based on CEGMA, out of the 458 core genes

Using a stringent threshold to remove genome scaffolds of potential bacterial or viral origin (Methods), we predict 35,913 and 26,609 high-quality gene models for *S. goreaui* and *S. kawagutii*, respectively (Supplementary Table 8). Usage profiles of codons and amino acids are shown in Supplementary Figs 4 and 5, respectively, and non-canonical splice sites in Supplementary Table 9 and Supplementary Fig. 6. Although we report fewer genes than in the published *Symbiodinium* genomes^12–14^, most (67.0 and 64.4% for *S. goreaui* and *S. kawagutii*, respectively) have transcriptome support; and we generally recovered more (Supplementary Fig. 7) of the 458 conserved core eukaryote genes (e.g. 436 in *S. goreaui* compared to 410 in the published *S. microadriaticum*^12^ based on TBLASTX; Supplementary Fig. 7C). Of these, 371 are common to all four *Symbiodinium* based on the predicted gene models (Fig. 1; Supplementary Data 1); similar results are observed for the corresponding genome sequences (Supplementary Fig. 7). About 94% of the predicted genes have introns, similar to *S. microadriaticum* (98.2%) and *Symbiodinium minutum* (95.3%); the earlier *S. kawagutii* genome assembly^13^ may have underestimated the proportion of intron-containing genes (Supplementary Table 8), due to a less-stringent approach to gene prediction. All coding sequences have higher G + C content (56.7% in *S. goreaui* and 55.0% in *S. kawagutii*) than does the genome overall, comparable to coding sequences from other *Symbiodinium* (57.7% in *S. microadriaticum* and 52.7% in *S. minutum*).

### Sequence divergence and synteny

Despite the seemingly high number of protein-coding genes, an earlier analysis of syntenic blocks^12^ found only several hundred blocks conserved in a pairwise manner among three published *Symbiodinium* genomes. Here we included our two new genome sequences in this analysis, and focused further on syntenic collinear blocks, requiring each block to contain the same genes in the same order and orientation with no gene losses (Methods). The *S. goreaui* and *S. kawagutii* genomes share the most collinear blocks with 889 blocks, implicating 8621 genes; 62 of these blocks are of size >15, with the largest containing 76 genes (Supplementary Table 10). Thus, substantial proportions of genes in these two genomes occur in clusters: for cluster size ≥5 genes, 32.4 and 24.0% of *S. kawagutii* and *S. goreaui* genes, respectively. These are likely to be underestimates, as the assemblies remain fragmentary. At the other end of the spectrum, the genomes of *S. microadriaticum* and *S. goreaui* share only 86 collinear blocks of size ≥ 5, with maximum size 12 and implicating 588 genes in total (Table 1; Supplementary Table 10). These results suggest that although Clades C and F are divergent, they are the most-closely related among the four analysed *Symbiodinium* genomes (in line with their phylogenetic relationship). They also suggest that C and F are more divergent from Clade A than from Clade B (in line with their phylogenetic relationship). Therefore, gene synteny supports and extends earlier conclusions, based on common marker sequences, about phylogenetic relationships among *Symbiodinium* clades^10,11^. The remarkable sequence divergence among *Symbiodinium* lineages (with <20% genome-sequence reads of *S. goreaui* and *S. kawagutii*, respectively, mapped to a genome of a different clade; Supplementary Fig. 1A) lends support to earlier observations^12,13^.

**Table 1:** Number of syntenic collinear blocks in *Symbiodinium* genomes

	*S. microadriaticum*, Clade A	*S. minutum*, Clade B	*S. goreaui*, Clade C
*S. minutum*, Clade B	370 (2816)	—	—
*S. goreaui*, Clade C	86 (588)	155 (1125)	—
*S. kawagutii*, Clade F	121 (893)	173 (1323)	889 (8621)

The number of identified syntenic collinear blocks for each pair of genomes (excluding self-comparisons) is shown, with the corresponding number of implicated genes in parentheses

### Genome duplication and evolution

**Fig. 2:**
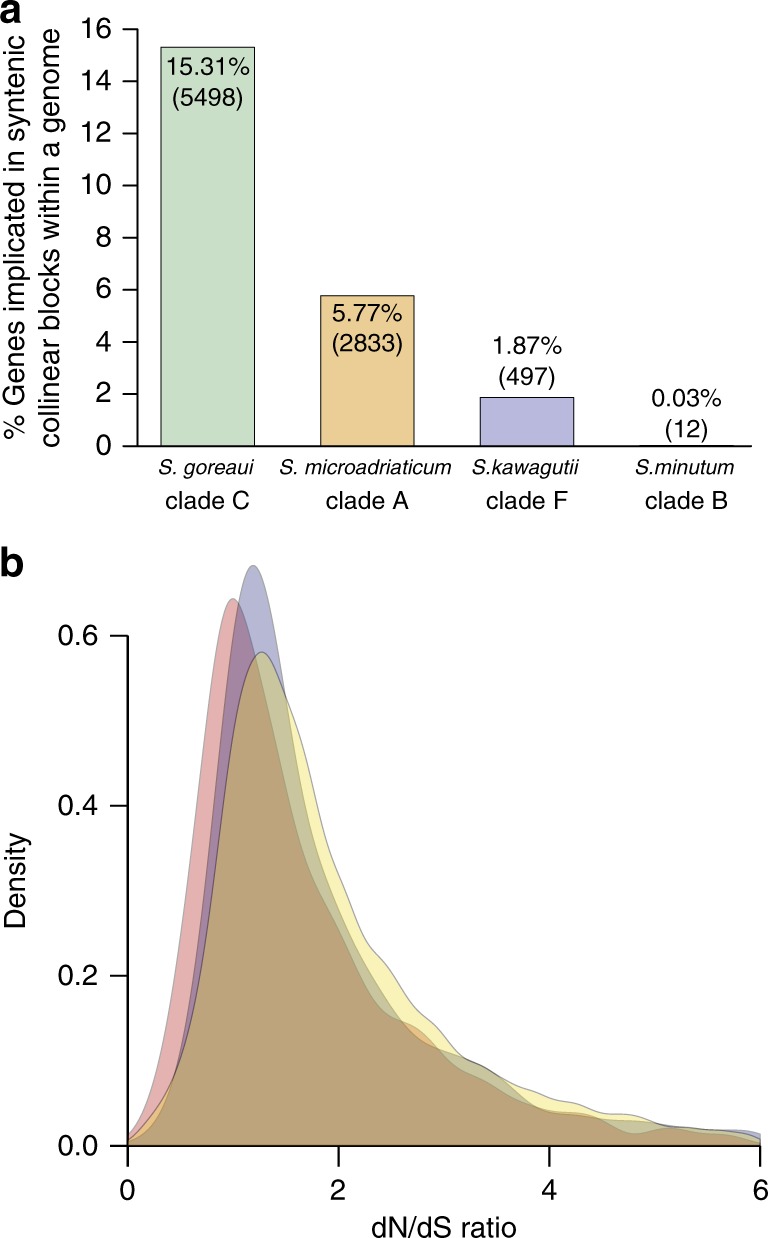
Genome duplication and evolution. **a** Percentage of genes that are implicated in syntenic collinear blocks within each genome as an indication of genome-fragment duplication. **b** The probability density of the dN/dS ratio for each pair of homologous genes found within syntenic collinear blocks in the genomes of *S. microadriaticum* (red: 1688 comparisons, mean 1.75, median 1.36), *S. goreaui* (yellow: 23499 comparisons, mean 2.04, median 1.65) and *S. kawagutii* (blue: 745 comparisons, mean 1.90, median 1.47). The *S. minutum* genome was excluded from this analysis due to incomplete data. Ratios between 0 and 6 are shown. The proportion of gene-pairs with dN/dS ratio >1 is 0.70–0.85 for these three genomes; the proportion of those with a ratio >6 is less than 0.02

To assess the extent of genome-fragment duplication in the *Symbiodinium* genomes, we further assessed the syntenic collinear blocks within each of the four *Symbiodinium* genomes (as opposed to those shared between two genomes; above); these blocks likely imply duplication of genome fragments. We recovered 3289 blocks implicating 5498 genes in the genome of *S. goreaui*, compared to 472 blocks (2833 genes) in *S. microadriaticum*, 121 blocks (497 genes) in *S. kawagutii*, and only 1 block (12 genes) in *S. minutum* (Fig. 2a); most of these blocks in *S. goreaui* and *S. kawagutii* contain genes annotated with metabolic functions (Supplementary Data 2). The draft genome of *S. minutum* covers only 616 Mbp of the estimated 1.5 Gbp genome^14^, thus the scarcity of collinear blocks within this genome is not surprising. While these results do not relate directly to whole-genome duplication, the genome of *S. goreaui* has the highest extent of genome-fragment duplication among the four, involving 15.31% (5498 of 35,913) of the predicted genes (Fig. 2a). This percentage compares to 5.77 and 1.87% in *S. microadriaticum* and *S. kawagutii*, respectively.

To assess the extent of adaptive selection of these duplicated genes within a genome, we further assessed the ratio (*ω*) of substitution rates in non-synonymous (dN) to synonymous (dS) sites^24^ between each pair of homologous genes located in the collinear blocks within a genome (Fig. 2b). Excluding *S. minutum* due to incomplete genome data, we observed the highest average *ω* in *S. goreaui* (2.04; based on 23,499 pairwise comparisons), followed by *S. kawagutii* (1.90; 745 comparisons), and *S. microadriaticum* (1.75; 1688 comparisons). Our mean/median results suggest that most of the duplicated genes have undergone positive selection (mean *ω* > 1; Fig. 2b), potentially leading to diversification of metabolic functions.

### Gene and protein functions

All annotated genes from *S. goreaui* and *S. kawagutii* genomes are listed in Supplementary Data 3 and 4, respectively. Of the 35,913 proteins predicted in *S. goreaui*, 31,646 (88.1%) show similarity (BLASTP, *E* ≤ 10^−5^) to sequences in UniProt; among these, 29,198 (81.3% of 35,913) and 19,718 (54.9%) are annotated with Gene Ontology (GO) terms or Pfam domains (Supplementary Table 11 and Supplementary Data 3). For *S. kawagutii*, 21,947 of 26,609 proteins (82.5%) find a match in UniProt (Supplementary Table 11 and Supplementary Data 4). *Protein kinase* (Pfam PF00069), *reverse transcriptase* (PF07727), *ion transport protein* (PF00520) and *ankyrin repeats* (PF12796) are among the most-abundant domains in *Symbiodinium*, appearing among the ten most abundant for each of the four genomes (Supplementary Table 12). Ankyrin repeat motifs are important in the recognition of surface proteins, and more generally in protein–protein interactions and have been implicated in mediating host–symbiont interactions across a variety of endosymbiotic associations^25^. Thus, proteins potentially involved in host–symbiont interaction (with phosphorylation, ion transport and protein recognition/interaction domains) are well represented in the predicted *Symbiodinium* proteomes. When these proteins were compared against those from *S. microadriaticum* and *S. minutum*, 35.1% of the identified homologous protein sets were recovered in all four genomes (Supplementary Fig. 7E).

We compared functions of proteins predicted from these four *Symbiodinium* genomes to a set of 27 eukaryotes (scoped more narrowly): 17 alveolates (ten other dinoflagellates, four ciliates, two apicomplexans and *Perkinsus marinus*), stramenopiles (two diatoms) and Archaeplastida (four rhodophytes, three chlorophytes and *Arabidopsis*). This 31-taxon set (1,136,347 proteins; Supplementary Tables 13 and 14) represents lineages in which one or more endosymbioses are implicated in plastid origin^26^; these proteins were clustered (based on sequence similarity) into 56,530 groups of size two or greater (Supplementary Table 14; see Methods). Using this 31-taxon dataset as background, we assessed the over-representation or under-representation of protein domains within our various groups of *Symbiodinium* proteins. We found 270 domains (Supplementary Data 5) to be significantly overrepresented (hypergeometric test, Benjamini-Hochberg^27^ adjusted *p* ≤ 0.05) in *Symbiodinium*. Interestingly, many domains e.g. *C-5 cytosine-specific DNA methylase* (PF00145), *planctomycete cytochrome c* (PF07635) and *sigma-70 region 2* (PF04542) of RNA polymerase are also overrepresented in the four *Symbiodinium* genomes in a similar comparison against 880,909 proteins in a 15-taxon set that includes ten other dinoflagellates and the immediate outgroup *Perkinsus marinus* (Supplementary Data 6). Therefore, compared to related eukaryotes and to other dinoflagellates, *Symbiodinium* is enriched in functions involved in methylation of cytosine, (photosynthetic) energy production and RNA polymerisation. Hydroxymethylation of uracil is common (12–70%) in dinoflagellate genomes^5^; while cytosine methylation has been described in *Symbiodinium*^28^, our findings suggest that cytosine methylation is more prominent in *Symbiodinium* than in these other dinoflagellates.

Activation of some retrotransposons is part of the stress-response mechanism in diatoms, plants and other eukaryotes^29^. The *reverse transcriptase* domain (PF07727) is enriched in *Symbiodinium* compared to both the 31-taxon and 15-taxon sets, suggesting that retrotransposition could be a prominent mechanism of stress response in *Symbiodinium* and dinoflagellates. Although we set a stringent threshold for removing putative bacterial or viral sequences (see Methods), 40 (~0.1%) of the final 41,289 genome scaffolds of *S. goreaui* have significant hits (BLASTN *E* ≤ 10^−20^) to the virus genomes^30^ isolated from the same *S. goreaui* (type C1) strain, with 16 identical regions (76–609 bp) distributed in nine scaffolds of lengths ranging from 1092 to 7,338,656 bp. Whether this indicates introgression of viral sequences remains to be determined.

### Positive selection of *Symbiodinium* genes

**Fig. 3:**
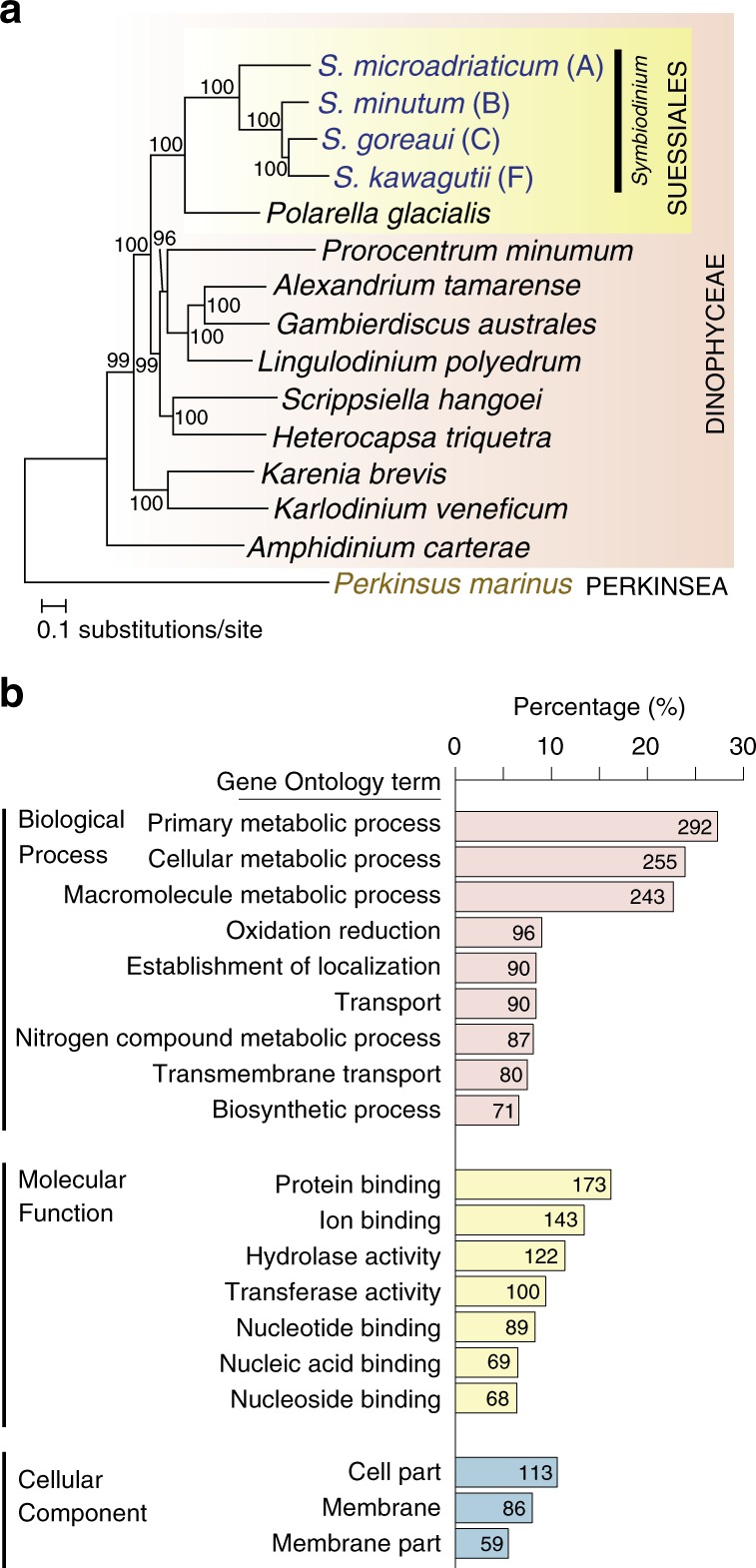
Testing for positive selection acting on *Symbiodinium* genomes. **a** The reference 15-species tree of *Symbiodinium*, dinoflagellates and *Perkinsus marinus* (as outgroup) based on single-copy orthologous genes, reconstructed based on a concatenated protein alignment with partition-specific maximum-likelihood model testing using IQtree, following Price and Bhattacharya^31^. Support based on 2000 rapid bootstraps is shown on each internal node, and the branch length is the number of substitutions per site. **b** Percentage of the 1069 positively selected gene sets in *Symbiodinium* that are annotated with GO (level 3) terms, shown for principal hierarchies Biological Process, Molecular Function and Cellular Component. The corresponding number of gene sets is shown on each bar

Using a branch-site model based on the ratio of dN/dS^24^ (Methods and Supplementary Fig. 8) and a reference species tree, we identified *Symbiodinium* genes showing evidence of positive selection in comparison to ten other dinoflagellates, with *P. marinus* as the outgroup (15 taxa: Supplementary Tables 13 and 14). The reference species tree (Fig. 3a) was computed following Price and Bhattacharya^31^, based on a concatenated protein alignment with partition-specific maximum-likelihood model testing (see Methods). We then based our analysis of adaptive evolution on all orthologous sets plus those homologous sets for which the protein tree is topologically congruent with our reference tree.

The 880,969 proteins from the 15-taxon set were first clustered into 310,617 homologous sets. We then adopted a stringent set of criteria (Supplementary Fig. 8) to filter these sets to yield the final 5675 sets: 1656 single-copy (orthologous) sets, and 4019 multi-copy sets for which the genus-level tree topology of each set, with *Symbiodinium* in an exclusive monophyletic clade, is congruent to the reference species phylogeny. Of the 5675 homologous sets, 2460 containing 7987 *Symbiodinium* proteins (5.0% of all 158,645 *Symbiodinium* proteins) show evidence of positive selection in one or more *Symbiodinium* lineages; 1069 of these sets are annotated with GO terms (Supplementary Data 7). Figure 3b shows the terms (level 3) in the three GO hierarchies that are shared by ≥5% of these 1069 sets. In the Biological Process hierarchy, metabolic processes are highly represented (*primary metabolic process* [292] and *macromolecule compound metabolic process* [243] are among the four most-frequent terms), followed by *oxidation reduction* [96] and transport (*establishment of localization* [90], *transport* [90], and *transmembrane transport* [80]). Highly represented terms in the Molecular Function hierarchy indicate binding of diverse molecules and ions, e.g. *protein binding* [173] and metabolism (*hydrolase* [390], *transferase* [344]). In Cellular Component, *cell part* [113], *membrane* [86] and *membrane part* [59] are the most frequent. Thus in *Symbiodinium* as represented by these four assemblies, broad aspects of metabolism, and transport including across membranes, show evidence of positive selection, in line with their recognised importance in cnidarian-dinoflagellate symbioses^12^.

We further assessed the enrichment of annotated GO terms among these 7987 *Symbiodinium* genes against all annotated terms in the four *Symbiodinium* genomes (Supplementary Data 8) in this study. Based on the enriched Biological Process terms, we observe four emergent themes among positively selected functions in *Symbiodinium* genes. The first theme is that functions associated with photosynthetic light reactions are enriched among the positively selected *Symbiodinium* genes; *photosynthesis, light reaction* and *photosystem II assembly* are significantly over-represented (Benjamini-Hochberg^27^ adjusted *p* ≤ 0.05), as are Cellular Component terms related to plastid functions e.g. *thylakoid*, *photosynthetic membrane*, *intracellular membrane-bounded organelle* (Supplementary Data 8). Coral bleaching has been associated with the loss of light-harvesting proteins and the subsequent inactivation of photosystem II (PSII) in *Symbiodinium* under combined light and temperature stress^32^. These earlier results suggest that coral bleaching associated with algal photobleaching can be ameliorated, at least in part, by thermal acclimation of *Symbiodinium* to improve the thermal tolerance of PSII. Therefore, these genes may have been selected to increase thermal resilience. Alternatively, this may reflect the adaptation of *Symbiodinium* to specific light and nutrient regimes imposed by symbiosis.

The second emergent theme involves the transport of ions and metabolites across membranes. *Intracellular transport*, *cytosolic transport*, *transition metal ion transport* and *copper ion transport* as well as terms related to transmembrane transport of amino acids, organic acids and carboxylic acids are significantly enriched (hypergeometric test, Benjamini-Hochberg^27^ adjusted *p* ≤ 0.05; Supplementary Data 8); these functions underpin multiple physiological processes, including but not limited to pH regulation, calcification and photosynthetic carbon fixation^33^. *Symbiodinium* investigated to date are enriched in bicarbonate and ammonium transporters compared with other dinoflagellates^12^. These biological processes are especially relevant to the maintenance and regulation of coral-dinoflagellate symbiosis^33^, possibly including its sensitivity and/or response to environmental stress.

The third theme is the enrichment of functions related to the biosynthesis and modification of amino acids and glycoproteins (Supplementary Data 8) e.g. *protein phosphorylation*, *peptide biosynthesis process*, *protein ADP-ribosylation*, *protein glycosylation*, *D-amino acid metabolic process* and *glycoprotein biosynthetic process*. Corals lack the capacity to synthesise a number of amino acids (e.g. cysteine in *Acropora digitifera*^34^), thus selection acting on the synthesis of amino acids may indicate the critical role of *Symbiodinium* in supplying amino acids both for self-preservation and for the coral host. Glycoprotein molecules are often surface-localised and in microbes form the basis of microbe-associated molecular patterns (MAMPs) which, in conjunction with a host-associated pattern recognition receptor, mediate recognition by a host^3^. Both in culture and *in hospite*, *Symbiodinium* exude glycoconjugates^3^. Where investigated, cell-surface glycan profiles are stable over time within a *Symbiodinium* culture, but can differ between clades within a host^35^. *N*-acetyl and mannosyl residues are prominent constituents of *Symbiodinium* cell-surface glycans, making them candidates for MAMPs that could participate in the establishment of symbiosis. Lin et al.^13^ reported a *S. kawagutii* glycan biosynthesis pathway distinct from that of *S. minutum*, again pointing to a possible role of glycans in specificity of host recognition^35^. Neubauer et al.^36^ demonstrated that the thrombospondin type 1 repeat (TSR) from the sea anemone *Aiptasia pallida* contains binding sites for glycosaminoglycan, and that blocking TSR led to decreased colonisation by *S. minutum*. Our results offer, to our knowledge, the first evidence of positive selection of functions underlying the biosynthesis and modification of amino acids and glycoproteins, suggesting that these functions are critical in the establishment of cnidarian-dinoflagellate symbioses.

Our fourth emergent theme relates to stress response. Enriched terms annotated for the positively selected genes include *cell redox homeostasis*, *translation initiation* and 22 terms describing the negative regulation of gene expression, transcription, RNA biosynthesis and cellular biosynthetic and metabolic processes (Supplementary Data 8). Environmental stressors can disrupt the cellular redox homeostasis and break down the coral-dinoflagellate symbiosis. Negative regulation of transcription may represent a stress response that safeguards the genome from accumulating DNA damage^37^; a similar stress response has been observed in coral^38^. Other enriched functions that may be related to stress response include *mitotic nuclear division*, *translation*, and various processes of nucleotide biosynthesis and modification e.g. *RNA methylation, rRNA methylation*, *DNA replication*, *RNA processing*, and *deoxyribonucleotide biosynthetic process*. Our results thus provide evidence that stress response is under positive selection in *Symbiodinium*, presumably (given their lifestyle) in relation to the establishment and/or maintenance of symbiosis.

### Do *Symbiodinium* have sex?

*Symbiodinium* have been hypothesised to reproduce sexually and to have a diploid life stage^39^, but definitive evidence for sex, e.g. karyogamy and meiosis, has yet to be observed^40^. The ability to reproduce sexually offers increased efficiency of selection and adaptation^41^. So far, the strongest evidence supporting meiotic potential in *Symbiodinium* comes from patterns of population-genetic variation revealed in allozymes, randomly amplified polymorphic DNA and other molecular markers^40,42^. Indeed, for some markers a higher degree of genetic variation has been observed in certain *Symbiodinium* clades than in dinoflagellates known to reproduce sexually^42^. More recently, differential gene expression analysis^43^ using a heterologous culture from which our sequenced *S. goreaui* was derived revealed an enrichment of gene functions related to meiosis under thermal stress, suggesting a switch from mitosis to meiosis under stress conditions.

**Fig. 4:**
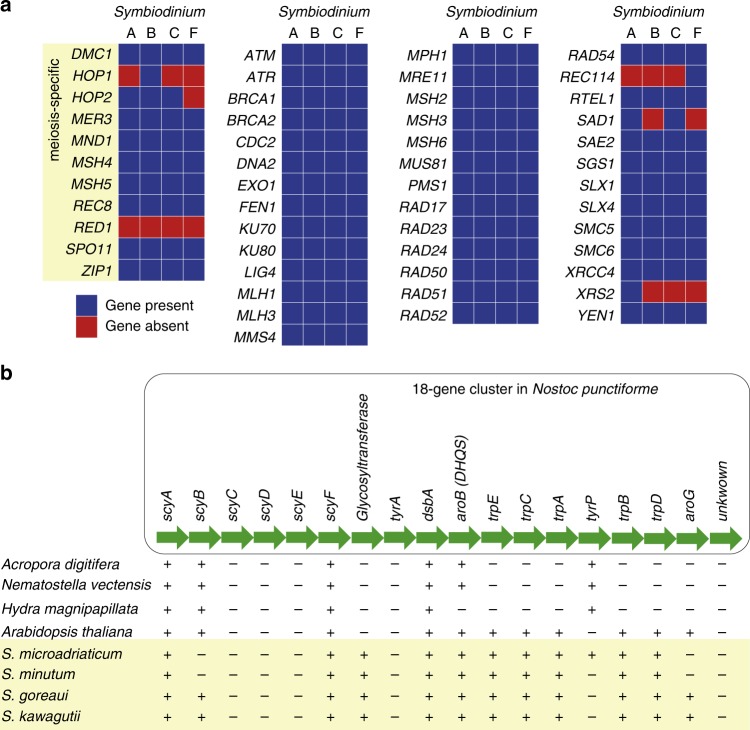
Recovery of genes in *Symbiodinium*. **a** Meiosis-related genes recovered in the genomes of *S. microadriaticum* (Clade A), *S. minutum* (Clade B), *S. goreaui* (Clade C) and *S. kawagutii* (Clade F). The first 11 genes are noted as meiosis-specific in Chi et al^45^. **b** Scytonemin biosynthesis genes in *Symbiodinium* genomes relative to the coral *Acropora digitifera*, sea anemone *Nematostella vectensis*, hydra (*Hydra magnipapillata*) and the green plant *Arabidopsis thaliana*. The order of the 18-gene cluster (shown in green arrows) in the cyanobacteria *Nostoc punctiforme* is used as a reference, with presence (+) and absence (−) of a gene in each species are shown. Figure modified from Shinzato et al^51^

Schurko and Logsdon^44^ described a meiosis detection toolkit, a set of 51 genes^45^ specific or related to meiosis that collectively point to a capacity for meiosis even in divergent or specialised eukaryotic genome. Incomplete genome coverage or assembly, sequence divergence, paralogy, patterns of overlapping function and evolutionary specialisation means that not all 51 need to be present or detectable for a lineage to be assessed as probably sexual, or only recently asexual^44^. Thirty-one of these genes were earlier identified in *Symbiodinium* Clades A and B^45^. Here, BLASTP search (*E* ≤ 10^−5^) of predicted proteins in these four *Symbiodinium* genomes recovered matches corresponding to 48 of the of 51 toolkit genes in *S. microadriaticum*, 47 in *S. minutum* and in *S. goreaui*, and 46 in *S. kawagutii* (Fig. 4a; Supplementary Data 9). Eight of the 11 meiosis-specific proteins were detected in all four *Symbiodinium*. REC114, SAD1 and XRS2 found weaker matches (*E* ≥ 10^−14^) in one to two genomes, although confirmatory UniProt domains were usually present (Supplementary Data 9). RAD17 is the *Schizosaccharomyces pombe* homolog of *S. cerevisiae* RAD24^46^, for which we find highly significant matches (*E* ≤ 10^−127^) in all four *Symbiodinium*. Moreover, 15 of the 51 genes show evidence of positive selection in *Symbiodinium* against other dinoflagellates (Supplementary Data 9). Our data imply that these four *Symbiodinium* are, or until recently have been, capable of meiosis.

### Response to light stress

Mycosporine-like amino acids (MAAs) are ultraviolet (UV)-protective compounds that, in corals and other marine organisms, also act as antioxidants scavenging reactive oxygen species. Up to five MAAs have been reported in *Symbiodinium* (Clades A, B and C) isolated from cnidarian hosts^47^. The MAA biosynthetic pathway involves dehydroquinate synthase (DHQS), *O*-methyltransferase (O-MT), an ATP-grasp and non-ribosomal peptide synthetase (NRPS)^48^. In cyanobacteria, a short-chain dehydrogenase may play a role in converting shinorine to palythine-serine^49^. Genes encoding these four MAA-biosynthetic enzymes were reported absent from the *S. kawagutii* genome^13^. Here, using known proteins in bacteria, fungi and cnidarians as queries, we recovered all five enzymes including the short-chain dehydrogenase from the *S. microadriaticum*, *S. goreaui* and *S. kawagutii* genomes (Supplementary Table 15); ATP-grasp was not found in *S. minutum*. These enzymes were earlier reported absent from *S. kawagutii*, and it was proposed that their absence can be compensated via coral-*Symbiodinium* co-evolution^13^; this hypothesis remains to be investigated, but we note that this *S. kawagutii* isolate has not been observed in association with an animal host^21^.

Scytonemin is a UV-blocker first reported in terrestrial cyanobacteria, and in contrast to MAAs was thought to be synthesised exclusively by cyanobacteria^48^. The genome of the cyanobacterium *Nostoc punctiforme* contains a UV-inducible 18-gene operon^50^ that specifies proteins of scytonemin biosynthesis and regulation, including proteins for the synthesis of aromatic amino-acid precursors such as chorismate. Homologs of six of these 18 genes have been described in the coral *Acropora digitifera*, and were considered putative instances of lateral genetic transfer^51^. We find 12 of these 18 genes in the genomes of *S. goreaui* and in *S. kawagutii*, 11 in *S. microadriaticum* and ten in *S. minutum* (Fig. 4b; Supplementary Table 16).

Genes responsible for biosynthesis of the aromatic amino acid tryptophan (*trpA*, *trpB*, *trpC*, *trpD* and *trpE*) and the two key enzymes of chorismate biosynthesis, *aroG* and *aroB* (dehydroquinate synthase, also important for MAA biosynthesis), are found in all *Symbiodinium* genomes, albeit so far in different scaffolds; these genes are also present in *Arabidopsis thaliana*, although not in corals or *Hydra* which, like most other animals, are unable to synthesise tryptophan. The recovery of more of these 18 genes in *Symbiodinium* than in corals or other animals (Fig. 4b) could reflect the impact of endosymbiotic association of ancestral cyanobacteria during the course of plastid evolution in photosynthetic eukaryotes^26^. The presence of multiple gene copies (Supplementary Table 16) also implicates genetic duplication. Our findings suggest that *Symbiodinium* has the capacity to produce scytonemin.

## Discussion

*Symbiodinium* can form associations with a wide range of cnidarian hosts (as well as some other marine invertebrates and protists) across broad geographic and temporal scales^11^. The symbiosis between corals and *Symbiodinium* relies on compatible host-symbiont recognition and sustainable nutrient exchange, both of which are vulnerable to external environmental factors including temperature and light. A sustainable coral-*Symbiodinium* association requires an adaptive capacity in the face of environmental extremes.

In this study, we generated the first draft genome of *S. goreaui* (Clade C), a much-improved draft genome of *S. kawagutii* (Clade F) and high-quality gene models for both. Comparative analysis revealed high divergence among the genomes of *Symbiodinium* from four clades, consistent with previous single-gene phylogenetic relationships. We found that many gene families related to the establishment and/or maintenance of symbiosis appear to be under positive selection in *Symbiodinium*, including genes related to photosynthesis, host-symbiont interactions and nutrient exchange.

In the absence of data from population genetics, the dN/dS ratio remains a valuable and widely used indicator of adaptive selection, including in host-symbiont relationships^52^. Artefacts and dS saturation may arise due to e.g. population size or structure, demographic history, gene flow, recombination or linkage, particularly when the ratio is computed within a population (or genome, as we do here); some of these artefacts can be avoided by use of branch-site models^53^. Moreover, *Symbiodinium* genomes may represent a favourable use case. Coral reef ecosystems have existed for ~240 M years^54^, individual reefs can be stable for thousands of years, and *Symbiodinium* can be transported over long distances in ocean currents^55^, potentially escaping local bottlenecks. In addition, *Symbiodinium* is haploid over much of its life history^56^, so deleterious alleles will be removed quickly.

We also identified complete, or near-complete, sets of genes indicative of the presence of meiosis and several mechanisms of stress tolerance, functions that have until now been considered absent from *S. goreaui* and *S. kawagutii*. *S. goreaui* (type C1) belongs to one of the most globally dominant clades (Clade C) on coral reefs; and analysis of its draft genome has provided important new insights into coral-algal symbiosis. This genomic resource is already demonstrating utility in the identification of symbiont fractions in de novo sequencing of coral tissues^57^, and will provide a foundation for targeted studies into the molecular biology and physiology of this crucial symbiosis and its adaptation to a changing environment.

## Methods

### Biological materials and DNA extractions

*Symbiodinium goreaui* (Clade C, type C1; AIMS-aten-C1-MI-cfu-B2, now AIMS culture collection SCF055-01) is a single-cell monoclonal culture first isolated from the coral *Acropora tenuis* at Magnetic Island (Queensland, Australia) at 3 m depth^58^; this culture is maintained at the Australian Institute of Marine Science, Townsville, Australia. Genomic DNA was extracted from these isolates using the Qiagen DNeasy Plant Mini Kit following the manufacturer’s protocol.

*Symbiodinium kawagutii* CS-156 (also known as CCMP2468) was first acquired from the Australian National Algae Culture Collection (ANACC). Unique cells were first selected under the microscope and grown in 24-well plates, from which unique cells were transferred onto agar plates. Their growth was monitored under the microscope to ensure colony formation before a colony was selected for further culturing in liquid medium. Throughout the experiment, the cells were cultured in f/2 medium containing ampicillin (100 μg mL^−1^), kanamycin (50 μg mL^−1^) and streptomycin (50 μg mL^−1^). PCR amplification using generic bacterial primers^59^ was performed regularly to identify potential bacterial contamination. High molecular-weight genomic DNA was extracted following the CTAB method described in Shoguchi et al.^14^.

### Generation and processing of sequencing data

For each isolate, sequence data (2 × 150 bp reads) were generated using multiple paired-end and mate-pair libraries on the Illumina HiSeq 2500 platform at the Australian Genome Research Facility, Melbourne. Details of insert length for each paired-end and mate-pair libraries are shown in Supplementary Table 1. Specifically, one of the paired-end libraries (of insert length 250 bp) was designed such that the read-pairs of 2 × 150 bp would overlap. In total, we generated 116.0 Gb (614.6 million reads) and 92.2 Gb (774.1 million reads) of sequence data for *S. goreaui* (type C1) and *S. kawagutii* (Clade F), respectively. Compared to *S. goreaui*, we generated fewer sequence data for *S. kawagutii* because some genome data of the same isolate^13^ are publicly available (see next section).

Adapter sequences were removed from the raw sequence data using Trimmomatic^60^, and erroneous bases were corrected using Quake^61^. For reads generated from the paired-end libraries, pairs with overlapping reads were merged into longer, single-end reads using BBMerge (http://jgi.doe.gov/data-and-tools/bbtools/bb-tools-user-guide/); we treated the other reads as bona fide paired-end reads. All reads generated from the mate-pair libraries were processed and classified using NXtrim^62^ based on read-orientation information (as observed based on the presence of adapter sequences in Nextera mate-pair libraries) into (a) paired-end, (b) single-end and (c) bona fide mate-pair reads. Due to the high standard deviation of estimated insert-lengths among the reads in (a), we treated both (a) and (b) as single-end reads. Details of all processed reads are shown in Supplementary Table 1.

### Comparative analysis of *S. kawagutii* genome sequence data

To ensure that the sequence data we generated for *S. kawagutii* CS-156 (=CCMP2468) were indeed from the same source as the published data^13^, we compared the sequence reads between the two data sources by mapping our reads onto the assembled genome in Lin et al.^13^, and conversely the reads in Lin et al.^13^ against our SPAdes genome assembly (see De novo genome assembly below), using CLC Genomics Workbench v7.5.1 (Qiagen). As shown in Supplementary Fig. 1A, about 89% of our reads mapped at high quality (MAPQ score ≥ 30) to the published genome assembly from Lin et al.^13^. In comparison, 96.0 and 87.2% of the reads respectively from our dataset and from Lin et al.^13^ mapped (MAPQ score ≥ 30) to our SPAdes genome assembly (Supplementary Fig. 1B). We recovered identical sequences of the phylogenetic marker genes (18S ribosomal RNA and internal transcribed spacer ITS2) from both genome datasets. To further assess assembly quality, we aligned the contigs from our preliminary genome assembly, and from the published assembly of *S. kawagutii*, against each of the ten fosmid sequences from Lin et al.^13^. Our SPAdes assembly has orders of magnitude fewer gaps and mismatches than the published assembly (Supplementary Fig. 2). In subsequent genome assemblies (below), we combined both published sequence reads from Lin et al.^13^ and our processed reads as a single dataset.

### De novo genome assembly

For each isolate we adopted a novel, integrative approach using multiple methods to assemble the genome de novo. First, to minimise assembly errors we systematically assessed the distances between read-pairs in all sequencing libraries. To do this, we first assembled all processed (single, paired-end and mate-pair) reads using CLC Genomics Workbench v7.5.1 (Qiagen) to generate an initial assembly; at this step, the insert-length information for each sequencing library was based on the estimate given by the sequencing provider. We then mapped all reads to the assembled contigs, and derived a more-accurate estimate of read-pair distances (i.e. via size of insert-length) for each sequencing library using *CollectInsertSizeMetrics* tool in Picard (https://broadinstitute.github.io/picard/).

Second, we assembled all processed reads using the more-accurate estimate of read-pair distances above, independently using (a) CLC Genomics Workbench v7.5.1 (Qiagen), (b) SPAdes^63^ and (c) ALLPATHS-LG^64^. For CLC and SPAdes, the contigs were further joined into longer scaffolds using mate-pair reads with SSPACE^65^; ALLPATHS-LG yielded genome scaffolds directly. Gaps within scaffolds were further filled using GapFiller^66^ at the default setting, thereby yielding three preliminary assemblies: the (a) CLC, (b) SPAdes and (c) ALLPATHS-LG assemblies (Supplementary Table 17). In addition to assembly statistics, we further assessed the quality of each assembly based on (a) full-length recovery of phylogenetic markers (18S ribosomal RNA and internal transcribed spacer region ITS2), (b) full-length recovery of coding sequences of known organellar genes, and (c) genome completeness based on conserved core eukaryote genes using CEGMA^67^ (Supplementary Data 1). As reference, we used all publicly available *Symbiodinium* ITS regions (both ITS1 and ITS2), mitochondrion-encoded genes and chloroplast-encoded genes in NCBI. While we recovered a high extent of CEGMA (eukaryote) genes (*S. goreaui*: 85.37%, *S. kawagutii*: 74.89%; Supplementary Table 17) in the SPAdes assemblies, these assemblies are highly fragmented (percentage of genome in scaffolds > 50 kb: 46.86% in *S. goreaui*, 74.89% in *S. kawagutii*). In comparison, we recovered a similarly high extent of CEGMA (eukaryote) genes (*S. goreaui*: 76.20%, *S. kawagutii*: 83.19% [the highest]; Supplementary Table 17) in the CLC assemblies that are more contiguous (percentage of genome in scaffolds > 50 kb: 73.64% in *S. goreaui*, 76.06% in *S. kawagutii*). The ALLPATHS-LG assemblies yielded the least number of scaffolds (thus higher contiguity; Supplementary Table 17), but many conserved genes and phylogenetic markers were misassembled (in fragments at multiple regions rather than in full-length). We therefore used the CLC assembly as the master assembly for each genome.

Third, we refined these master assemblies using MUMmers in GMCloser^68^ by filling the gaps and merging scaffolds using contigs from the SPAdes and ALLPATHS-LG assemblies, followed by another step of gap-filling using GapFiller^66^. This gave us the refined master assemblies.

### Identification and removal of bacterial and viral sequences

To identify putative bacterial and viral sequences in the genome scaffolds of *S. goreaui* and *S. kawagutii*, we followed the approach of Aranda et al.^12^ with some modifications. In brief, we first searched the scaffolds (BLASTN) against a database of bacterial and viral genomes (see “Methods”), and identified those with hits at bit score >1000 and *E* ≤ 10^−20^; we considered these as significant hits. We then examined the sequence cover of these regions in each scaffold, and identified the percentage (in length) contributed by these regions relative to the scaffold’s full length. Aranda et al.^12^ used a threshold of 50% sequence cover as indication of putative bacterial or viral contaminant, and thus removed scaffolds containing >50% of putative bacterial or viral regions. Here, we systematically assessed the number of implicated genome scaffolds across the different thresholds of percentage sequence cover of putative bacterial or viral regions, and the corresponding gene models in these scaffolds (Supplementary Fig. 9). At the most-stringent threshold (0% sequence cover), any scaffold with any significant bacterial or viral hits is considered a contaminant, here 333 and 90 scaffolds in *S. goreaui* and *S. kawagutii*, respectively (Supplementary Fig. 9A, B); these represent <1% of the total assembled scaffolds in each genome. In contrast, at the lenient threshold of 90% sequence cover, only 32 and 2 scaffolds, from *S. goreaui* and *S. kawagutii*, respectively, are considered contaminants. In both genomes, the number of scaffolds shows a sharp decrease from thresholds at 0 to 10% sequence cover, followed by a gradual decrease as the subsequent thresholds become less stringent. A similar trend is observed with the implicated gene models on these scaffolds (Supplementary Fig. 9C and D). The 0% threshold may be too strict in these cases, since bacterial-like genes are known to be present in dinoflagellates. Here we chose 10% as the deciding threshold, i.e. any scaffold with significant bacterial or viral hits covering >10% of its length was considered a contaminant. In this way 129 and 33 scaffolds (and the gene models implicated within) were removed from *S. goreaui* and *S. kawagutii*, respectively.

### Genome annotation and gene prediction

We adopted a comprehensive ab initio approach for gene prediction using all available dinoflagellate proteins, as well as all *Symbiodinium* genes and transcriptomes, as guiding evidence. For each genome assembly, a de novo repeat library was first derived using RepeatModeler (http://www.repeatmasker.org/RepeatModeler/). All repeats (including known repeats in RepeatMasker database release 20150807) were masked using RepeatMasker (http://www.repeatmasker.org/).

We used transcriptome data to guide functional annotation of assembled genomes. For *S. goreaui*, we used the published transcriptome data (NCBI accession GSE72763) from Levin et al.^43^. For *S. kawagutii*, we used the transcriptome data of CCMP2468 (MMETSP0132; RNA-Seq reads after filtering for adapters and low-quality reads) available from MMETSP^69^, and the published transcripts (generated using the 454 platform) from Lin et al.^13^. For RNA-Seq data, we assembled the reads using Trinity^70^ independently in “de novo” mode and “genome-guided” mode, after which vector sequences were trimmed using SeqClean (https://sourceforge.net/projects/seqclean/) based on UniVec database (ftp://ftp.ncbi.nlm.nih.gov/pub/UniVec/; build v9.0).

We used a customised PASA^71^ script (available at http://smic.reefgenomics.org/download/) that recognises an additional donor splice site (GA), and used the program alongside TransDecoder^71^ to predict coding sequences (CDS) in each genome. These CDS were searched (BLASTX, *E* ≤ 10^−20^) against a customised protein database that consists of RefSeq proteins release 78 and other annotated or predicted *Symbiodinium* proteins (total of 49,732,862 sequences; Supplementary Table 18). Only near full-length CDS were included in the subsequent analysis; here we require these CDS to have a near full-length alignment (>70%) to a protein in the database, using a script provided with Trinity.

The near full-length gene models were checked for TEs using HHblits^72^ (probability = 80% and E-value = 10^−5^) searching against the JAMg transposon database (https://sourceforge.net/projects/jamg/files/databases/), as well as with Transposon-PSI (http://transposonpsi.sourceforge.net/). Gene models containing TEs were removed from the gene set, and redundancy reduction was conducted using CD-HIT^73^ (ID = 75%). The remaining gene models were processed using the Prepare_golden_genes_for_predictors.pl (http://jamg.sourceforge.net/) script from the JAMg pipeline (altered to recognise GA donor splice sites). This script produces a set of “golden genes”, which were used as a training set for the gene-prediction packages AUGUSTUS^74^ and SNAP^75^. We used a customised code of AUGUSTUS (available at http://smic.reefgenomics.org/download/) so it recognises GA donor splice sites, and trained it to predict both coding sequences and untranslated regions; SNAP was trained for both GT and GC donor splice sites. Soft-masked genomes were passed to GeneMark-ES^76^ for training and gene prediction.

UniProt-SwissProt (release 2016_06) proteins, MMETSP Suessiales proteins and the predicted *Symbiodinium* proteins (above) were clustered using CD-HIT (ID = 100%). The clustered proteins were used to produce a set of gene predictions using MAKER^77^ with protein2genome; the custom repeat library was used by RepeatMasker as part of MAKER prediction. A primary set of predicted genes was produced using EvidenceModeler^78^, which had been altered to recognise GA donor splice sites. This package combines the gene predictions from PASA, SNAP, AUGUSTUS, GeneMark-ES and MAKER protein2genome, as well as the masked repeats (using custom repeat library), into a single set of evidence-based predictions. The weightings used for the package were: PASA 10, Maker protein 8, AUGUSTUS 6, SNAP 2 and GeneMark-ES 2. The final genome assemblies, predicted gene models and proteins are available at http://refuge2020.reefgenomics.org/.

We adopted multiple approaches to assess genome completeness. Established methods including CEGMA^67^ and BUSCO^79^ are based on conserved genes in a limited number of eukaryote model organisms that are distantly related to dinoflagellates. The use of these methods resulted in relatively low recovery of conserved eukaryote genes in *Symbiodinium* (e.g. 33–42% of BUSCO genes; Supplementary Fig. 7B) when run at default setting. We further assessed completeness using BLAST based on predicted proteins from the gene models and the assembled genome scaffolds. For each genome, we searched (BLASTP, *E* ≤ 10^−5^)against the predicted proteins using the 458 CEGMA proteins^67^. We also searched against the CEGMA proteins using the genome scaffolds (BLASTX *E* ≤ 10^−5^), against genome scaffolds using the 458 CEGMA proteins (TBLASTN, *E* ≤ 10^−5^), and against genome scaffolds using the 458 CEGMA transcripts (TBLASTX, *E* ≤ 10^−5^) (Supplementary Data 1 and Supplementary Fig. 7).

### Analysis of genome synteny and collinearity

Using all predicted genes and their associated genomic positions, we assessed the number of syntenic collinear blocks (i.e. regions with the same genes coded in the same order, free from rearrangement or loss) shared pairwise among genomes of *S. microadriaticum* (Clade A)^12^, *S. minutum* (B)^14^, *S. goreaui* (C) and *S. kawagutii* (F). We used BLASTP (*E* ≤ 10^−5^) to search for similar proteins between each pairwise genomes for inter-genome comparisons, and to search for similar proteins within each genome for self-genome (within-genome) comparisons. Next we used MCScanX^80^ with parameter –s 5 to sort the BLASTP matches (alignments) based on genomic positions; to minimise the number of collinear gene pairs arising from tandem repeats, we report only collinear blocks that consist of five or more genes.

### Analysis of plastid genomes

Plastid genomes of dinoflagellates occur as minicircles. Here we focused on our ALLPATHS-LG genome assemblies. To identify putative plastid genome fragments in our genome data, we used plastid gene sequences identified in *Symbiodinium* type C3^81^, *Symbiodinium minutum*^82^ and *Heterocapsa triquetra*^7^ as queries in BLASTN searches against our genome assemblies. To identify the conserved core regions in the putative plastid genome sequences, we set a high mismatch penalty (match score = 1, mismatch scores = −4, gap opening cost = 5, and gap extension cost = 2, *E* ≤ 10) in reciprocal BLASTN searches. The identified core region was then used to identify other genome scaffolds that were not previously identified by alignment with known plastid-encoded genes. These scaffolds were searched against the NCBI’s non-redundant nucleotide database (BLASTN at default parameters) to assess if they align to any known genes. All scaffolds identified as being of plastid origin, both those encoding known plastid genes and those encoding only core regions, were checked for circularisation using pairwise BLASTN (*E* ≤ 10^−10^). Artemis^83^ and Artemis Comparison Tool (ACT)^84^ were used to annotate the isolated scaffolds. The putative plastid genome sequences and their annotation are available at http://refuge2020.reefgenomics.org/.

### Analysis of mitochondrial genomes

Mitochondrial genes from the dinoflagellates *Alexandrium catenella* and *Karlodinium micrum* were used as queries to identify putative mitochondrial genome fragments within our ALLPATHS-LG assemblies using BLASTN (*E* ≤ 10^−10^). Nucleotide sequences of the *cox1, cox3* (cytochrome oxidase subunits 1 and 3 of complex IV) and *cob* (cytochrome *b* of complex III) genes and fragments of the large subunit rRNA (LSU rRNA) and the small subunit rRNA (SSU rRNA) were retrieved from the NCBI non-redundant nucleotide database. Scaffolds with *cox1*, *cox3* and *cob* hits were considered putative mitochondrial genome fragments, and were assessed for evidence of circularisation using pairwise BLASTN. The putative mitochondrial genome sequences and their annotation are available at http://refuge2020.reefgenomics.org/.

### Functional annotation of gene models

We adopted a similar approach to Aranda et al.^12^ to annotate gene models based on sequence similarity searches against known protein sequences. Protein sequences predicted using the standard genetic code were used as query (BLASTP, *E* ≤ 10^−5^) first against Swiss-Prot, and those with no Swiss-Prot hits subsequently against TrEMBL (both databases from UniProt release 2016_10). GO (http://geneontology.org/) terms associated with Swiss-Prot and TrEMBL hits were obtained using the UniProt-GOA mapping (release 2016_10).

### Identification of homologous protein sets and gene families

Our workflow for delineation of sets of putatively homologous proteins, multiple sequence alignment, generation of protein-family and reference trees, and analysis of selection is shown in Supplementary Fig. 8. Protein sequences were generated computationally, using the standard genetic code, from genome and/or transcriptome sequences of 31 organisms including *Symbiodinium* (Supplementary Table 13; 31-taxon set). Similarly, a 15-taxon set (14 dinoflagellates and the outgroup *Perkinsus marinus*) was established. Sequences of length <30 amino acids were removed, and sets of putatively homologous proteins were generated using OrthoFinder^85^. Sets that contain ≥ 4 proteins, including at least one from a *Symbiodinium*, were taken forward. We assumed that all proteins within each set (and thus the corresponding coding genes) share a common ancestor. We considered sequences within single-copy sets (i.e. those in which each genome is represented no more than once) to be orthologs. Those in multi-copy sets may include co-orthologs and/or paralogs. We refer to sets that contain proteins only from *Symbiodinium*, plus the *Symbiodinium* singletons, as *Symbiodinium*-specific. For enrichment analysis of annotated features (GO terms or Pfam domains), we compared the features within the *Symbiodinium*-specific set against those in each background set (i.e. the 31-taxon set and, separately, the 15-taxon set below) using a hypergeometric test; features with Benjamini-Hochberg^27^ adjusted *p* ≤ 0.05 were considered significant.

### Analysis of positive selection in *Symbiodinium* genes

For this analysis, we focus on homologous protein sets from the 15-taxon dataset. For the 15-taxon set we sorted the 310,617 protein sets into 1656 single-copy (ortholog) and 16,301 multi-copy sets. Multiple sequence alignments were carried out using MAFFT^86^ v7.245 at -linsi mode; questionably aligned columns and rows were removed from these alignments using trimAl^87^ with the -automated1 option.

Branch-site models (BSMs; see below) require a reference topology. We follow Price and Bhattacharya^31^ to generate a maximum-likelihood (ML) reference species tree using single-copy protein sets. The trimmed single-copy protein alignments were concatenated prior to ML inference of the species phylogeny using IQTREE^88^; each alignment represents a partition for which the best evolutionary model was determined independently. Support for each node was assessed using 2000 rapid bootstraps. The species tree so generated (Fig. 3a) is similar to that of Price and Bhattacharya^31^, with very strong support (≥96% bootstrap replicates) for all internal nodes; the *Symbiodinium* and Suessiales (*Symbiodinium* + *Polarella glacialis*) clades received 100% bootstrap support.

Of all trimmed protein alignments, those with ≥60 aligned positions and ≥4 sequences were used in subsequent analysis. A total of 1656 single-copy protein sets satisfied these criteria. For multi-copy protein sets, we imposed further filtering criteria. We first inferred individual ML trees for the multi-copy sets using IQ-TREE, and each resulting protein tree was compared with the reference species tree. Those congruent with the reference species tree at genus level, and in which all *Symbiodinium* are resolved as an exclusive monophyletic clade, were judged paralog-free and used in subsequent BSM analysis (Supplementary Fig. 8). Among the 16,301 multi-copy sets of the 15-taxon analysis, 1656 (10.2%) resolve all *Symbiodinium* sequences into an exclusive monophyletic clade and are topologically congruent at genus level with the reference species tree (i.e. contain co-orthologs but not paralogs) and were retained, while the remaining 14,645 failed one or both of these filtering criteria (i.e. contain presumed paralogs) and were not analysed further (Supplementary Fig. 8). The percentages of missing data and parsimoniously informative sites in all 5675 filtered protein alignments for the 15-taxon set are detailed in Supplementary Data 10. For each filtered alignment, we used the corresponding coding-sequence alignment (codon alignment) generated using PAL2NAL^89^ in the BSM analysis. Some predicted protein sequences in MMETSP^69^ do not match their corresponding CDS, sometimes due to problematic translation and other times due to a frameshift. For these, we used MACSE^90^ to derive the codon alignments.

We applied the BSM implemented in the *codeml* program in PAML 4.9^91^ to detect positive selection signal unique to the *Symbiodinium* lineage. BSMs allow the dN/dS ratio (*ω*) to vary among both sites and branches, making it possible to infer selection at both. We computed two models: a null model with fixed *ω* = 1, and an alternative model that estimates *ω* in our defined foreground branches (here, the node that leads to all *Symbiodinium* lineages). We then compared the likelihoods of these two models to determine the better fit. To reduce false positives we applied *q*-value estimation for false discovery rate control, as implemented in R package *qvalue* to adjust *p* values. Instances with an adjusted *p* ≤ 0.05 were considered significant.

We also performed gain-and-loss analysis on the gene sets corresponding to the protein sets under a Dollo parsimony model^92^, using *dollop* as implemented in PHYLIP 3.69 (http://evolution.genetics.washington.edu/phylip/). Here we focused on the *Symbiodinium* subtree (i.e. lineages for which genome data are available) with the immediate outgroup of *Polarella glacialis*. To assess the impact of Markov clustering granularity in OrthoFinder on our results, we analysed gene gain and gene loss using homologous protein sets that were generated independently using the inflation parameter *I* at 1.0, 1.5 and 2.0 (see Supplementary Note 1 and Supplementary Fig. 10).

### Data availability

All sequence data generated from this study are available at the NCBI Short Read Archive (SRA) BioProject accession PRJEB20399, with SRA accessions ERS1940397 (for *S. goreaui*), and ERS1940392, ERS1940393, ERS1940394, ERS1940395 and ERS1940396 (for *S. kawagutii*). Assembled genomes, predicted gene models and proteins are available at http://refuge2020.reefgenomics.org/.

### Code availability

The customised scripts for AUGUSTUS and PASA used in this study were previously published in Aranda et al.^12^; they are available at http://smic.reefgenomics.org/download/.

## Electronic supplementary material

Supplementary Information

Description of Additional Supplementary Files

Supplementary Data 1

Supplementary Data 2

Supplementary Data 3

Supplementary Data 4

Supplementary Data 5

Supplementary Data 6

Supplementary Data 7

Supplementary Data 8

Supplementary Data 9

Supplementary Data 10
