# Publisher Correction: *Symbiodinium* genomes reveal adaptive evolution of functions related to coral-dinoflagellate symbiosis

**DOI:** 10.1038/s42003-018-0117-4

**Published:** 2018-08-23

**Authors:** Huanle Liu, Timothy G. Stephens, Raúl A. González-Pech, Victor H. Beltran, Bruno Lapeyre, Pim Bongaerts, Ira Cooke, Manuel Aranda, David G. Bourne, Sylvain Forêt, David J. Miller, Madeleine J. H. van Oppen, Christian R. Voolstra, Mark A. Ragan, Cheong Xin Chan

**Affiliations:** 1 Institute for Molecular Bioscience, The University of Queensland, Brisbane, QLD 4072 Australia; 2 Australian Institute of Marine Science, Townsville, QLD 4810 Australia; 3 ARC Centre of Excellence for Coral Reef Studies, James Cook University, Townsville, QLD 4811 Australia; 4 Department of Molecular and Cell Biology, James Cook University, Townsville, QLD 4811 Australia; 5 Global Change Institute, The University of Queensland, Brisbane, QLD 4072 Australia; 6 Institute for Biodiversity Science and Sustainability, California Academy of Sciences, San Francisco, CA 94118 USA; 7 Red Sea Research Center, Division of Biological and Environmental Science and Engineering, King Abdullah University of Science and Technology (KAUST), Thuwal, 23955-6900 Saudi Arabia; 8 College of Science and Engineering, James Cook University, Townsville, QLD 4811 Australia; 9 Research School of Biology, Australian National University, Canberra, ACT 2601 Australia; 10 School of BioSciences, The University of Melbourne, VIC, 3010 Australia; 11 School of Chemistry and Molecular Biosciences, The University of Queensland, Brisbane, QLD 4072 Australia; 12 Present Address: Laboratoire d’excellence CORAIL, Centre de Recherches Insulaires et Observatoire de l’Environnement, Moorea, 98729 French Polynesia

Correction to: *Communications Biology*; 10.1038/s42003-018-0098-3; published online 17 July 2018

In the original HTML version of the paper, the following affiliation was missing for author Cheong Xin Chan: School of Chemistry and Molecular Biosciences, The University of Queensland, Brisbane, QLD 4072, Australia. This affiliation was incorrectly assigned to author Pim Bongaerts as “Present Address”. All affiliations were published correctly in the PDF version of the paper and have now been corrected in the HTML.

