# Supplementary material for: Symbiodinium genomes reveal adaptive evolution of functions related to coral-dinoflagellate symbiosis
Source: Commun Biol. 2018 Jul 17;1:95. doi: 10.1038/s42003-018-0098-3 (PMC6123633; doi:10.1038/s42003-018-0098-3)
Supplement: Supplementary file 1 — Supplementary Information [file 42003_2018_98_MOESM1_ESM.pdf]

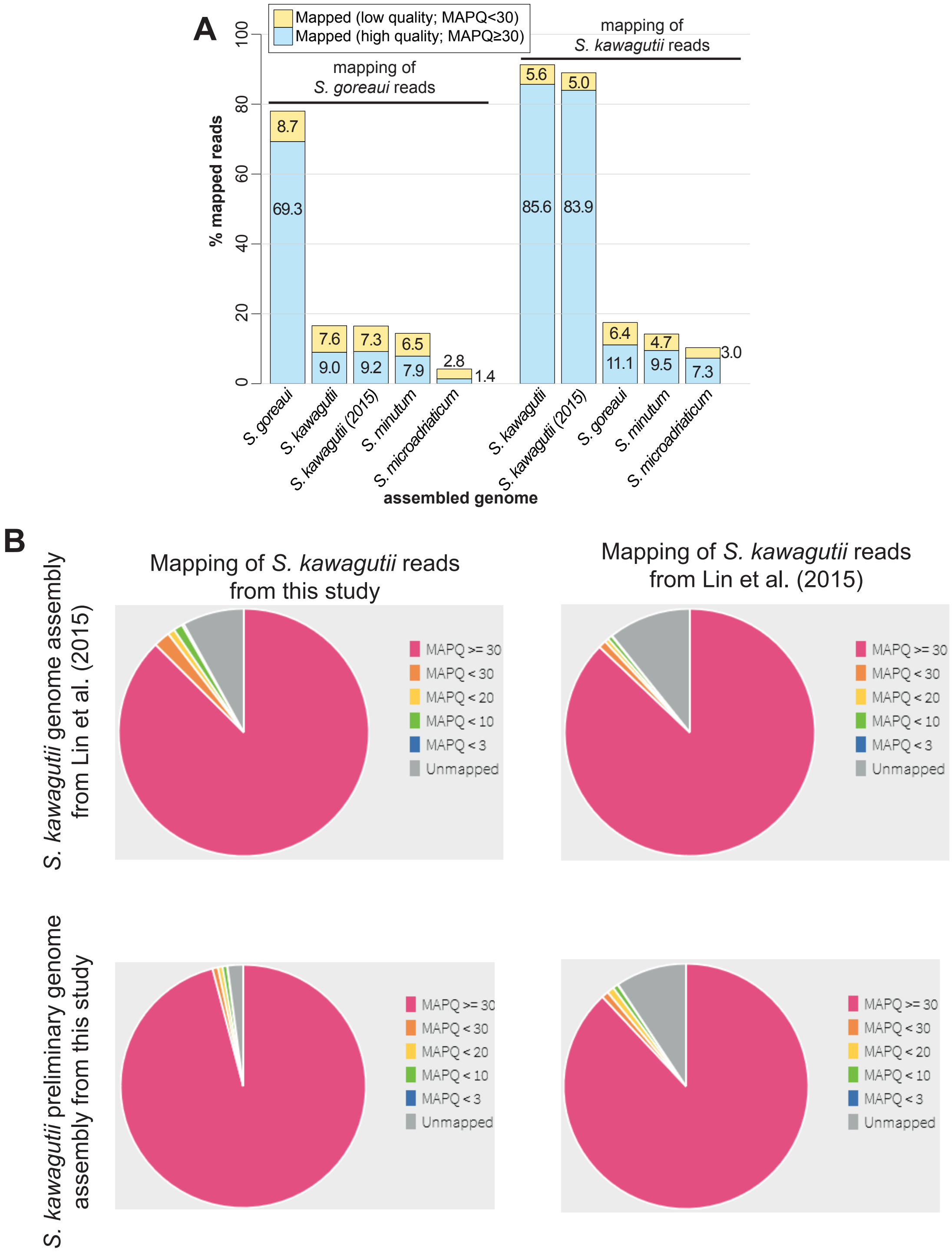

Supplementary Figure 1. (A) Mapping of sequence reads from *S. goreaui* and *S. kawagutii* to five assembled *Symbiodinium* genomes: *S. goreaui* and *S. kawagutii* from this study, *S. kawagutii* (Lin et al. 2015), *S. minutum* (Shoguchi et al. 2013), and *S. microadriaticum* (Aranda et al. 2016). (B) Mapping of *S. kawagutii* sequence reads from this study (left) and from Lin et al. (2015; right) onto the assembled genome from Lin et al. (2015; above), and the preliminary genome assembly from this study (bottom). Mapping at various thresholds of mapping quality (MAPQ) are shown.

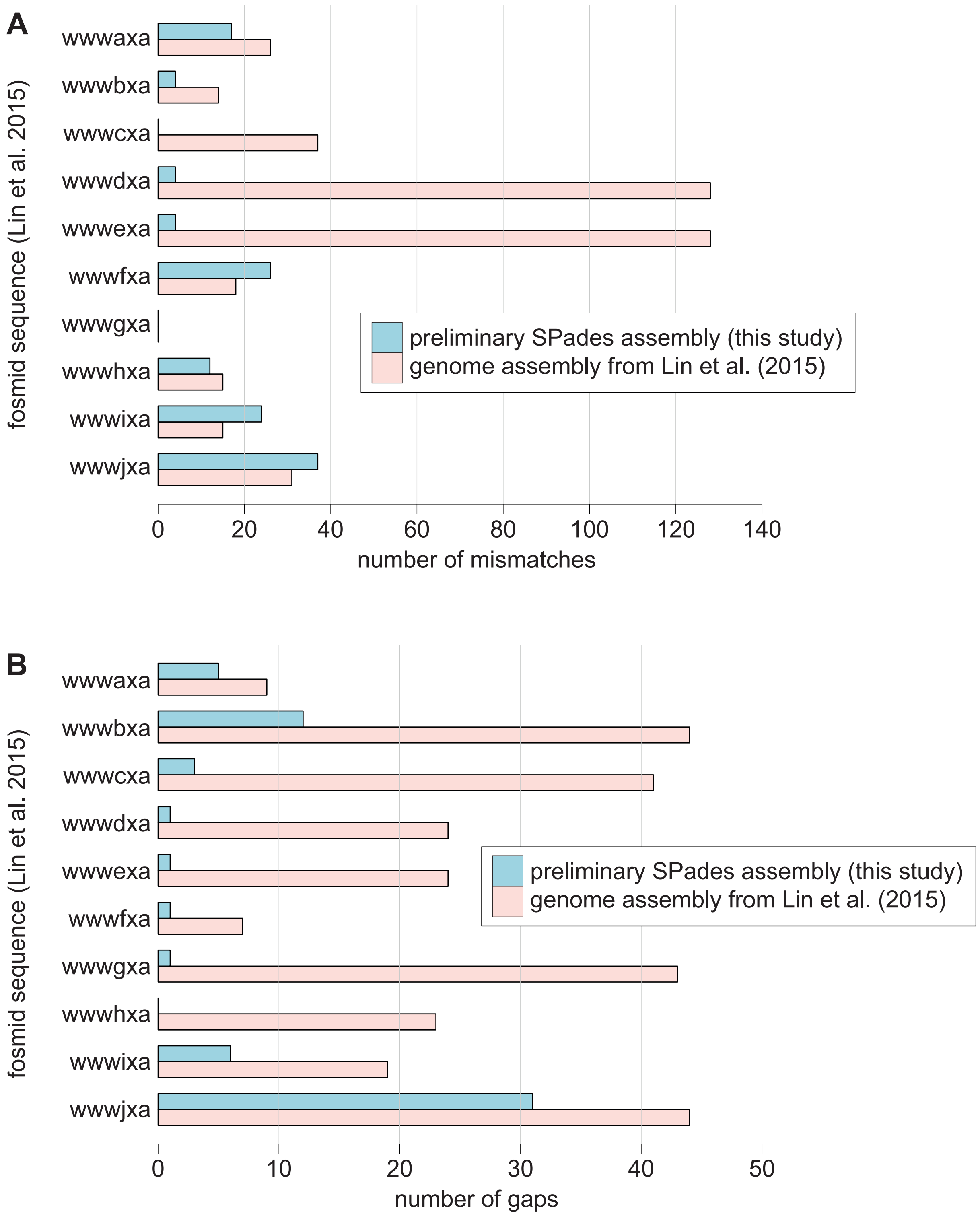

Supplementary Figure 2. Mapping of ten *S. kawagutii* fosmid sequences from Lin et al. (2015) onto the assembled genome from Lin et al. (2015) and the preliminary SPAdes genome assembly from this study, based on observed (A) number of mismatches and (B) number of gaps in the aligned regions.

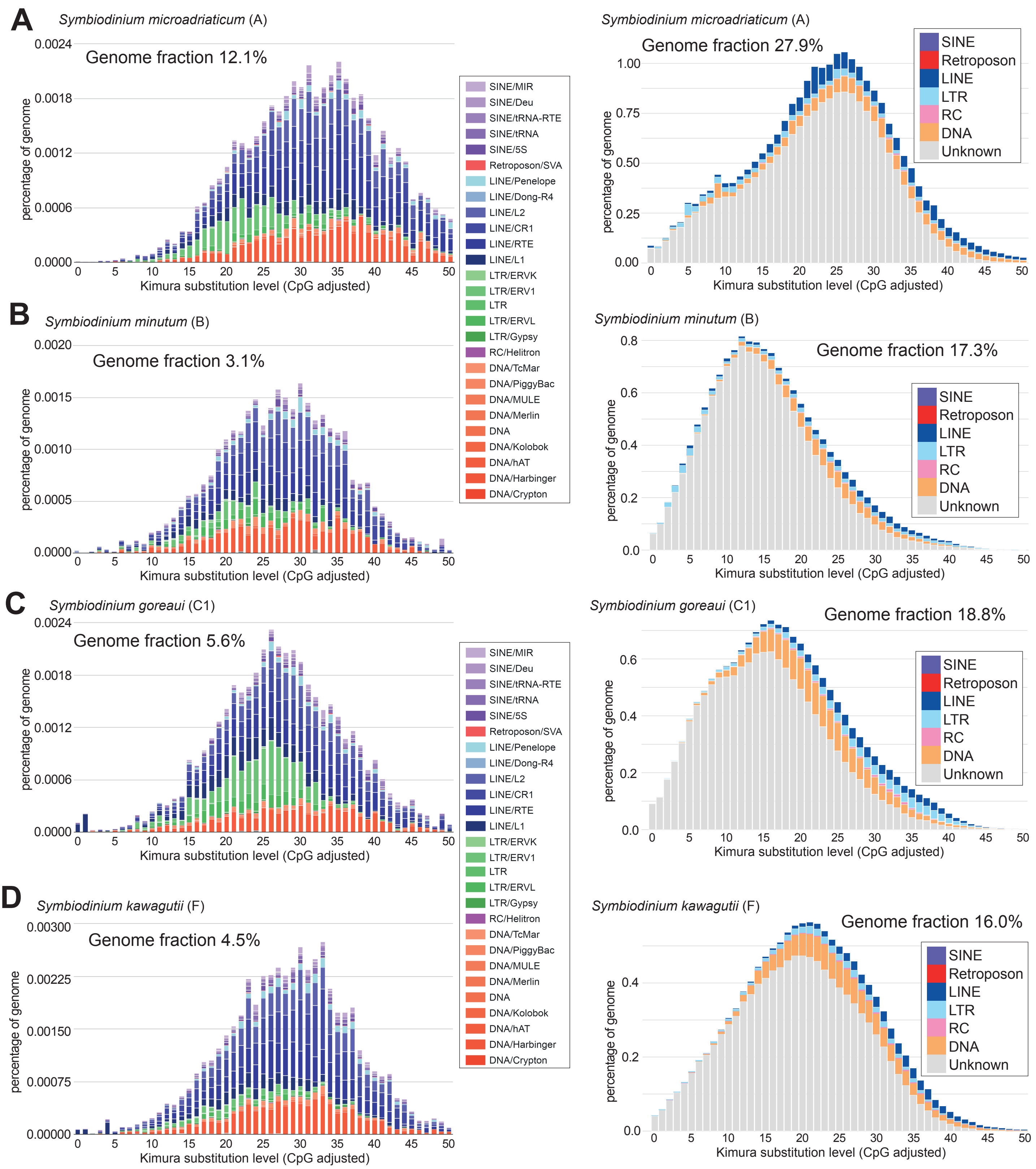

Supplementary Figure 3. Interspersed repeat landscape for each genome of (A) *S. microadriaticum*, (B) *S. minutum*, (C) *S. goreaui* and (D) *S. kawagutii*, shown for only known repetitive elements on the left, and for all repetitive elements (including unknown repeats) on the right.

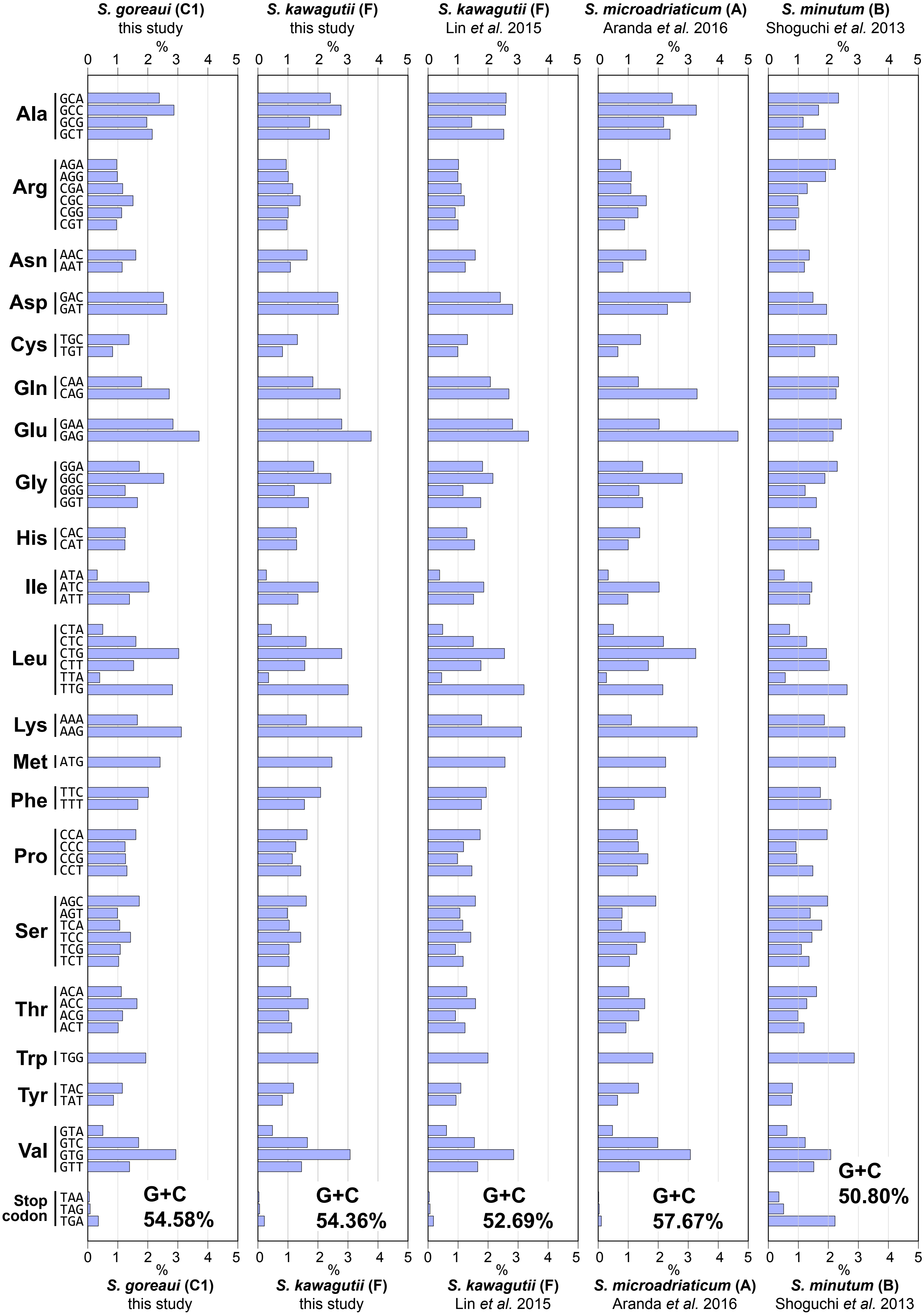

Supplementary Figure 4. Codon usage profiles based on predicted gene models in *S. goreaui* (this study), *S. kawagutii* (this study), *S. kawagutii* (Lin et al., 2015), *S. microadriaticum* and *S. minutum*.

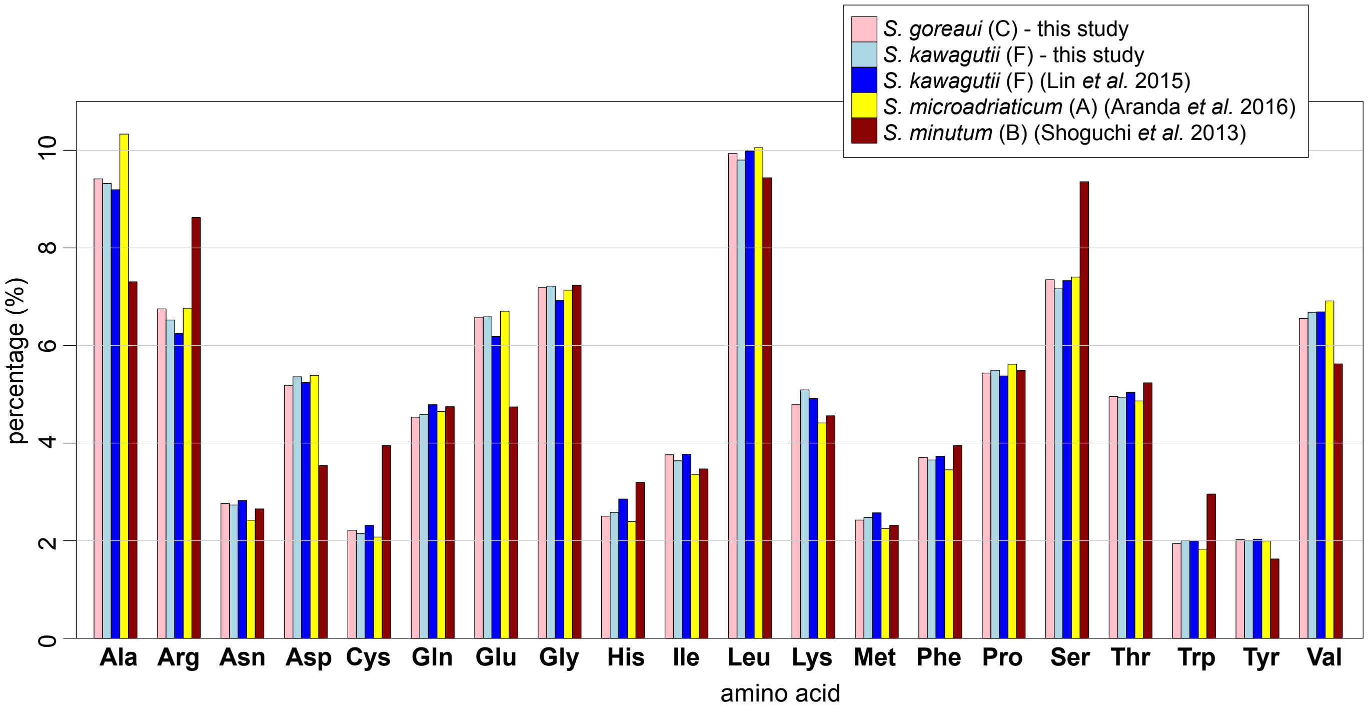

Supplementary Figure 5. Amino acid profiles based on predicted gene models in *S. goreaui* (this study), *S. kawagutii* (this study), *S. kawagutii* (Lin et al., 2015), *S. microadriaticum* and *S. minutum*.

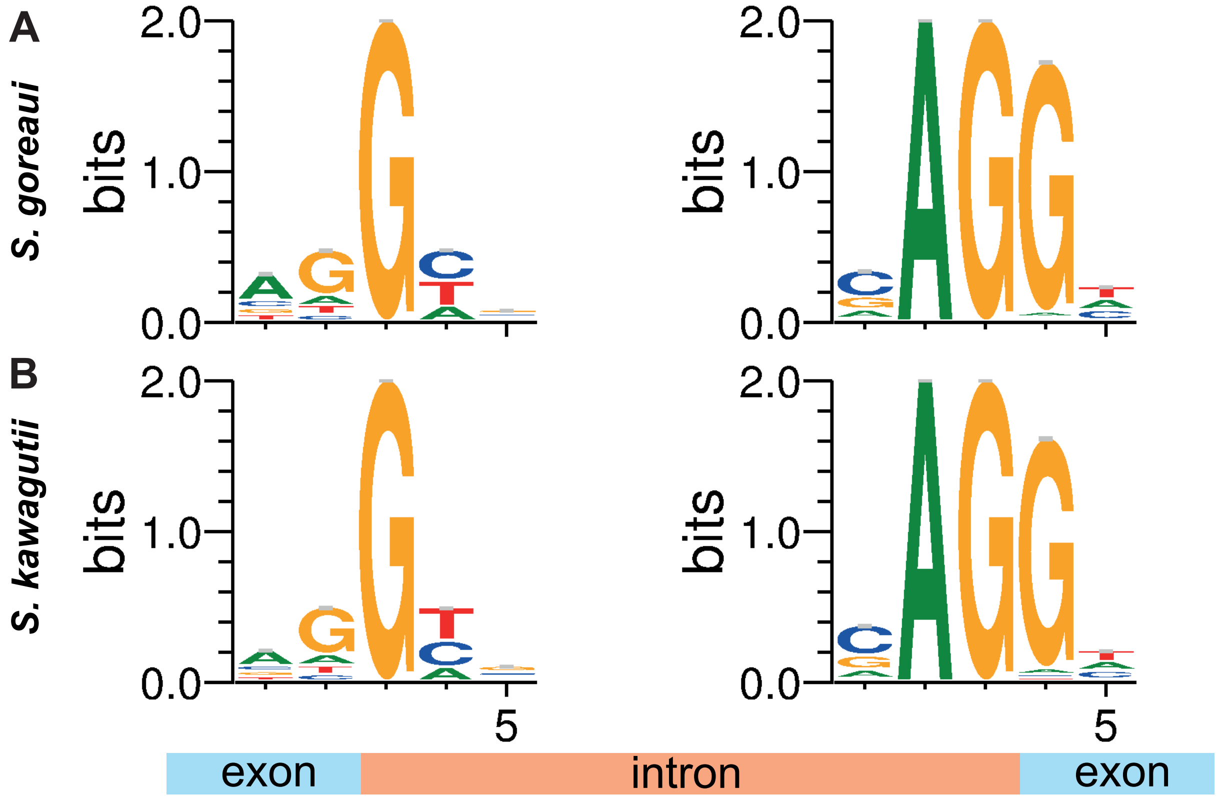

Supplementary Figure 6. Non-canonical splice sites in the genomes of *S. goreaui* (A) and *S. kawagutii* (B), shown for the donor (left) and the acceptor (right).

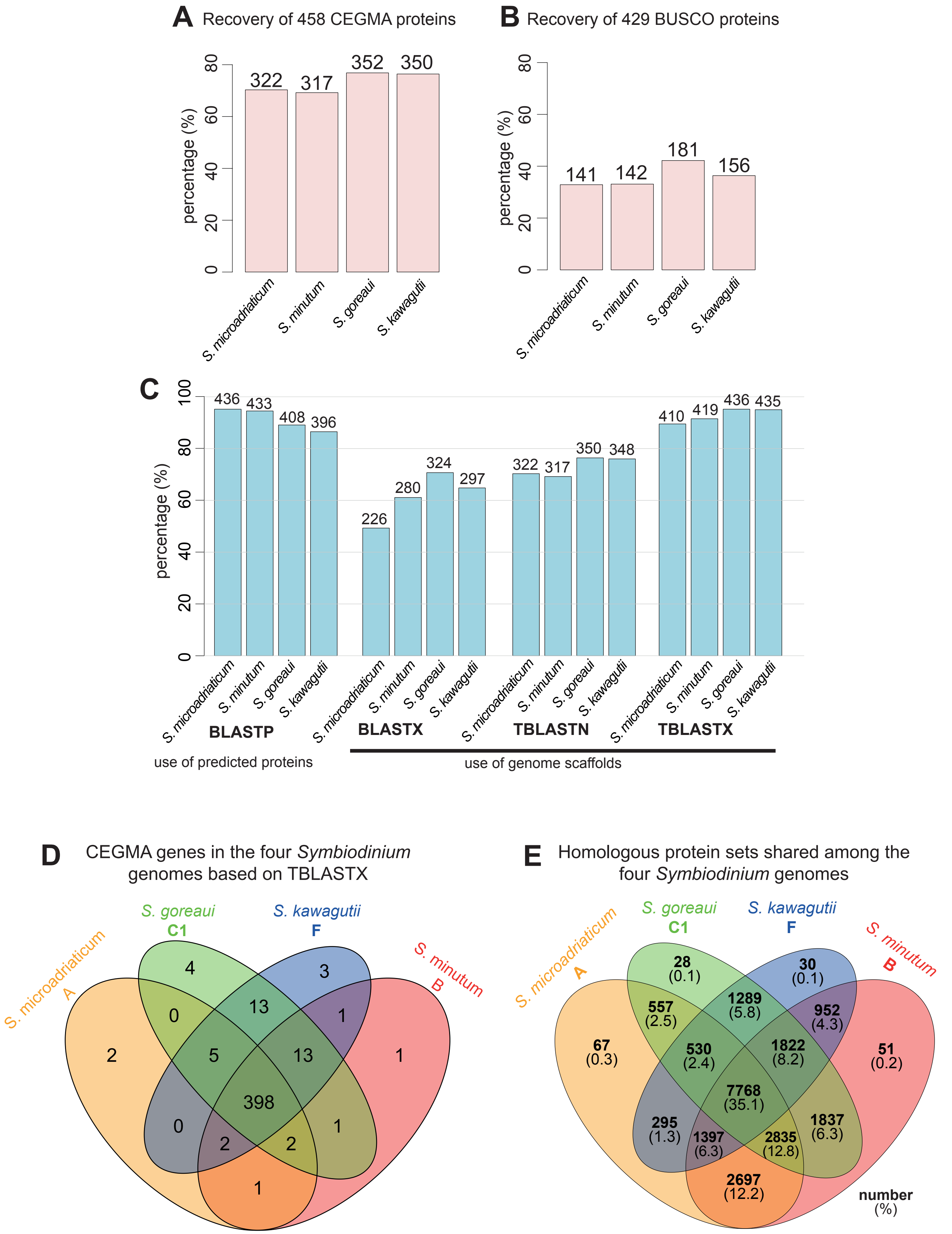

Supplementary Figure 7. Recovery of (A) CEGMA and (B) BUSCO genes in *Symbiodinium* using the default programs, and the recovery of CEGMA genes based on (C) a suite of BLAST analyses using the predicted proteins of *Symbiodinium* and their genome scaffolds. (D) The overlapping CEGMA genes in each genome based on TBLASTX analysis. (E) Number and percentage of homologous protein sets shared among the four *Symbiodinium* genomes.

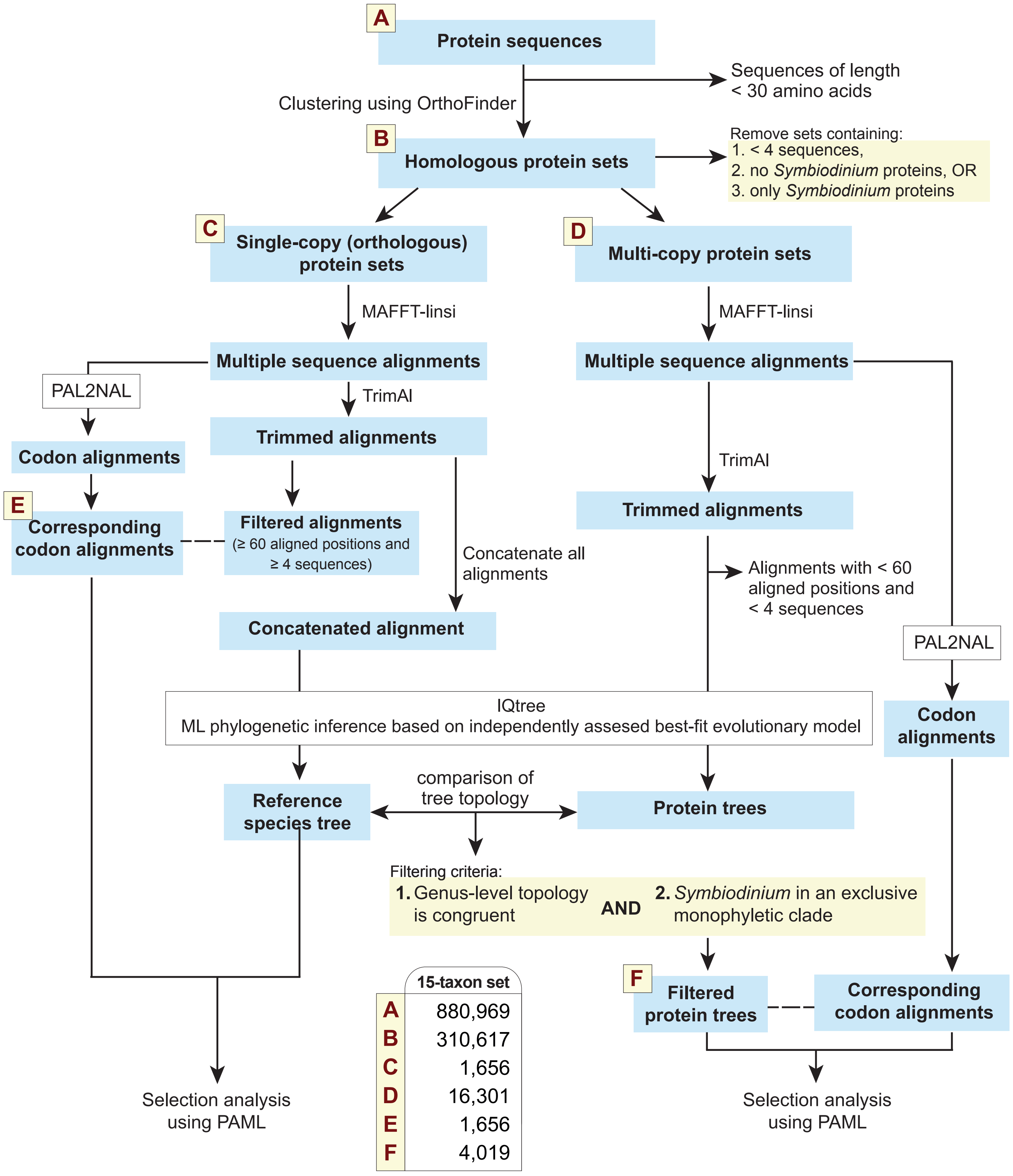

Supplementary Figure 8. Schematic workflow for analysis of positive selection in this study, with number of items at the six designated stages (A, B, C, D, E and F) shown for the analyses using the 15-taxon set.

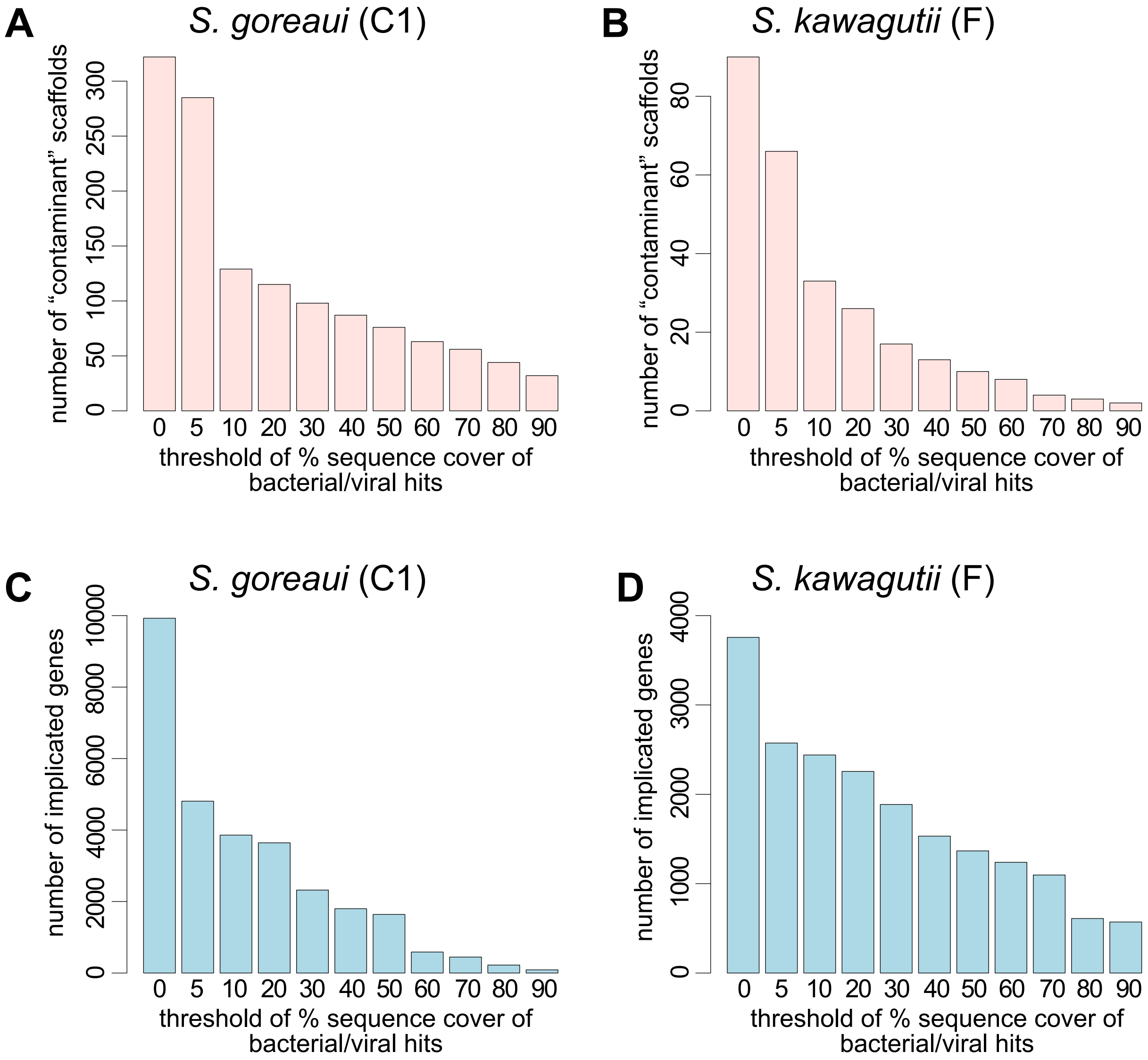

Supplementary Figure 9. Number of "contaminated" genome scaffolds that contain regions with hits to bacterial or viral genomes, shown at different threshold of percentage sequence cover in the scaffolds for (A) *S. goreaui* and (B) *S. kawagutii*. The implicated gene models in the corresponding scaffolds in *S. goreaui* and *S. kawagutii* are shown in (C) and (D) respectively.

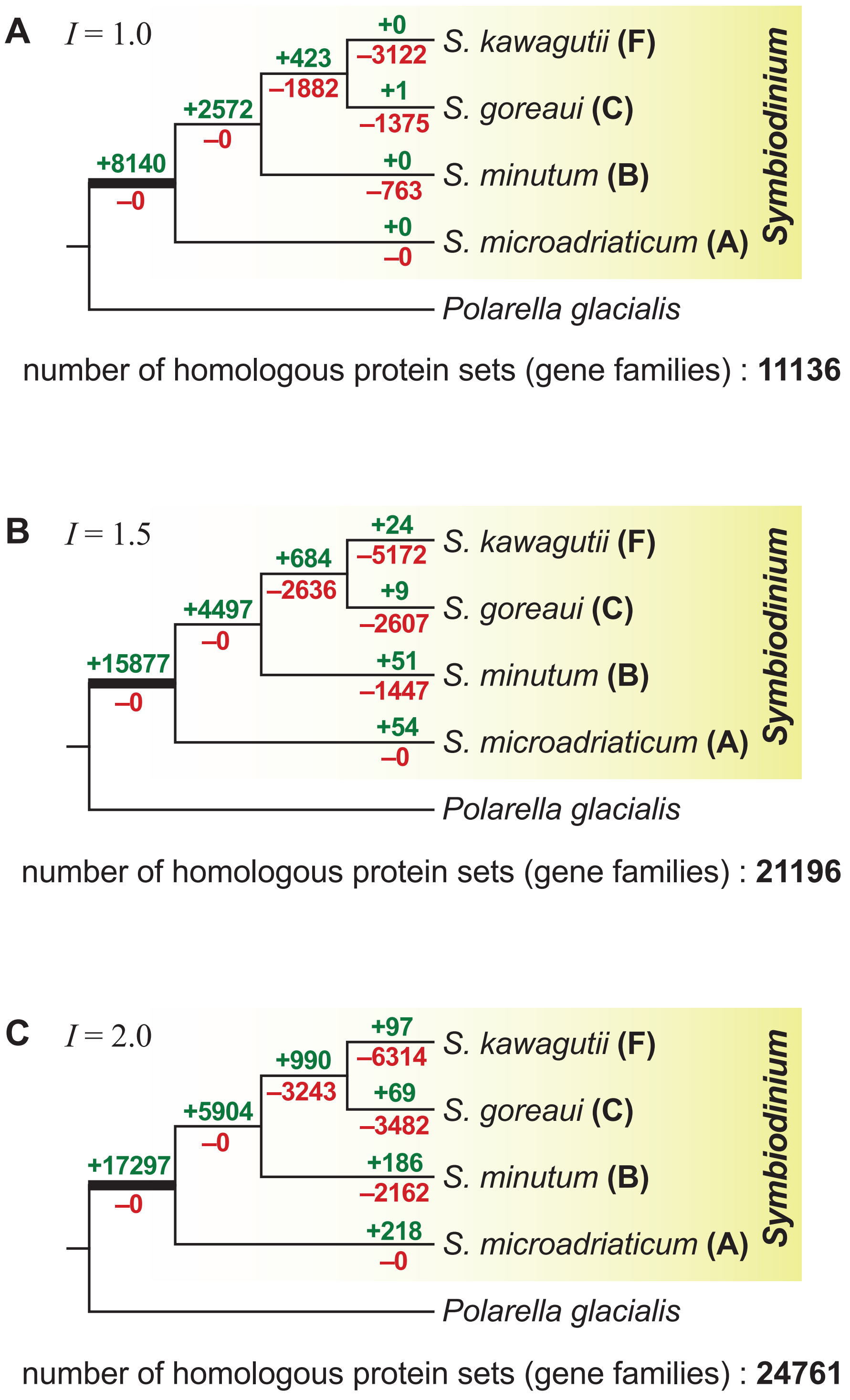

Supplementary Figure 10. Analysis of gene gain and gene loss in distinct *Symbiodinium* lineages based on clustering of gene families using inflation parameter *I* at (A) 1.0, (B) 1.5 and (C) 2.0.

Supplementary Table 1. New genome sequencing data generated in this study.

Library	Insert size (bases)	Number of raw 150-base reads	Total bases of raw reads	Number of filtered reads	Total bases of filtered reads
***Symbiodinium goreaui* SCF055-01 (clade C)**					
Paired-end-230-read1	230	114,097,328	17,114,599,200	112,131,983	16,673,830,321
Paired-end-230-read2	230	114,097,328	17,114,599,200	111,484,222	16,460,439,867
Paired-end-500-read1	500	143,659,886	21,548,982,900	143,659,886	21,025,889,473
Paired-end-500-read2	500	143,659,886	21,548,982,900	143,659,886	19,917,972,952
Mate-pair-3k-read1	3000	51,818,233	7,772,734,950	19,716,458	2,338,880,924
Mate-pair-3k-read2	3000	51,818,233	7,772,734,950	19,267,699	2,273,478,043
Mate-pair-6k-read1	6000	46,780,528	7,017,079,200	18,079,228	2,139,909,539
Mate-pair-6k-read2	6000	46,780,528	7,017,079,200	17,651,099	2,079,770,820
Mate-pair-9k-read1	9000	30,718,808	4,607,821,200	10,903,776	1,303,946,153
Mate-pair-9k-read2	9000	30,718,808	4,607,821,200	10,655,510	1,269,966,863
Single-end (after filtering)	n/a	n/a	n/a	78,528,939	5,183,436,887
**TOTAL**		**774,149,566**	**116,122,434,900**	**685,738,686**	**90,667,521,842**
***Symbiodinium kawagutii* CS-156 (=CCMP2468; clade F)**					
Paired-end-230-read1	230	175,549,642	26,332,446,300	171,450,581	25,504,756,026
Paired-end-230-read2	230	175,549,642	26,332,446,300	171,450,581	25,153,088,954
Mate-pair-4k-read1	4000	51,491,804	7,723,770,600	19,529,439	2,335,641,250
Mate-pair-4k-read2	4000	51,491,804	7,723,770,600	19,529,439	2,326,433,751
Mate-pair-6k-read1	6000	52,197,110	7,829,566,500	19,803,840	2,368,759,747
Mate-pair-6k-read2	6000	52,197,110	7,829,566,500	19,803,840	2,357,239,118
Mate-pair-9k-read1	9000	28,050,295	4,207,544,250	10,846,597	1,288,094,198
Mate-pair-9k-read2	9000	28,050,295	4,207,544,250	10,846,597	1,282,668,909
Single-end (after filtering)	n/a	n/a	n/a	178,881,831	2,347,886,305
**TOTAL**		**614,577,702**	**92,186,655,300**	**622,142,745**	**64,964,568,258**

Supplementary Table 2. Statistics of genome assembly.

	Symbiodinium goreaui **SCF055-01 (clade C, type C1)**	*Symbiodinium kawagutii* **CS-156 (=CCMP2468, clade F)**
%G+C	44.83	45.72
Total number of scaffolds	41,289	16,959
Total assembled bases (bp)	1,027,792,016	1,048,482,934
N50 length of scaffolds (bp)	98,034	268,823
Maximum scaffold length (bp)	8,337,000	5,159,000
Total number of contigs	267,890	109,980
N50 length of contigs (bp)	6,576	35,743
Maximum contig length (bp)	3,449,000	956,496
Number of scaffolds > 50 Kb	6,160	4,204
% genome in scaffolds > 50 Kb	72.87	88.91
% gap	13.22	15.24
Estimated genome size (bp)	1,189,354,686	1,072,636,679

Supplementary Table 3. Estimation of genome size for *S. goreaui* and *S. kawagutii* based on *k*-mer coverage.

Estimate of genome size (bp)		
k	***Symbiodinium goreaui* SCF055-01 (clade C, type C1)**	***Symbiodinium kawagutii* CS-156 (=CCMP2468, clade F)**
17	1,155,783,960	1,060,560,949
19	1,176,285,240	1,075,387,053
21	1,194,377,556	1,057,483,567
23	1,210,721,782	1,069,035,022
25	1,192,813,515	1,080,674,525
27	1,206,146,062	1,092,678,958
Mean	1,189,354,686	1,072,636,679

Supplementary Table 4. Putative plastid genome fragments in *S. goreaui* and the encoded genes. Evidence of circularisation and number of core regions identified are shown.

**Sequence**	**Encoded gene**	**Sequence length (bp)**	**Circular**	**Number of core regions identified**
SC1_Plastid_1	*psbC/23S rRNA/ psaA/atpB*	10,928	No	4
SC1_Plastid_2	*psbB/psbA*	4,954	No	2
SC1_Plastid_3	*16S rRNA/petB*	4,255	No	3
SC1_Plastid_4	*psaB*	2,253	No	1
SC1_Plastid_5	*atpA*	2,048	No	1
SC1_Plastid_6	*psbD*	1,781	No	1
SC1_Plastid_7	*petD*	1,476	No	1
SC1_Plastid_8	*psbE*	1,006	No	1
SC1_Plastid_9	*psbI*	2,349	No	1
SC1_Plastid_10	none ("empty")	2,394	No	1

Supplementary Table 5. Putative plastid genome fragments in *S. kawagutii* and the encoded genes. Evidence of circularisation and number of core regions identified are shown.

Sequence	Encoded gene	Sequence length (bp)	Circular	Number of core regions identified
SF_Plastid_1	*psaB*	11,097	No	1
SF_Plastid_2	*psbA/atpB*	6,627	No	3
SF_Plastid_3	*psaA*	7,802	Yes	2
SF_Plastid_4	*psbC*	4,497	No	2
SF_Plastid_5	*atpA*	4,489	Yes	0
SF_Plastid_6	*23S rRNA*	3,059	No	0
SF_Plastid_7	*psbB*	2,237	No	1
SF_Plastid_8	*psbD*	1,500	No	0
SF_Plastid_9	*petD*	1,290	No	0
SF_Plastid_10	*petB*	1,132	No	0
SF_Plastid_11	*16S rRNA*	1,070	No	0
SF_Plastid_12	*16S rRNA*	1,696	Yes	1
SF_Plastid_13	*psbI*	1,581	No	0
SF_Plastid_14	none ("Empty")	1,517	Yes	2
SF_Plastid_15	none ("Empty")	1,293	No	1

Supplementary Table 6. Length and GC content of the coding and non-coding regions identified in the plastid genome sequences in *Symbiodinium*.

	S. goreaui **(clade C)**	***S. kawagutii*** **(clade F)**	***Symbiodinium*** **clade C3 (Barbrook et al. 2014)**
Total Length (bp)	33,444	50,887	27,303
Total GC%	41.35%	38.88%	41.84%
**Coding regions**			
Total length (bp)	15,393	23,753	15,828
G+C (%)	36.80	35.46	36.03
**Non-coding regions**			
Total length (bp)	18,051	27,134	11,475
G+C (%)	45.64	42.56	48.22

Supplementary Table 7. Core conserved regions identified in plastid genomes of *S. goreaui* and *S. kawagutii*.

**Species**	**Core conserved region**	**Length (bp)**
S. goreaui	TAATGGGCTGGGTGCCCTACCCAGCCCATATGGGCCCACGCTTCG CGGGGCCCATAACGGCCCTTCGGGCCTTCAAAAA	79
S. kawagutii	GTGATTCCCAAGGACCGGAAGCCGGACCTTGGGAATCAGCG	41

Supplementary Table 8. Statistics of predicted gene models in *Symbiodinium* genomes.

	S. microadriaticum	***S. minutum***	***S. kawagutii***	***S. goreaui***	***S. kawagutii***
Symbiodinium clade	**A**	**B**	**F**	**C**	**F**
Reference	Aranda et al. 2016	Shoguchi et al. 2013	Lin et al. 2015	**This study**	**This study**
**Assembly**					
Total assembled bases (bp)	808,242,489	609,476,485	935,067,369	1,027,792,016	1,048,482,934
G+C content (%)	50.51	43.46	43.97	44.83	45.72
**Genes**					
Number of genes	49,109	47,014	36,850	35,913	26,609
Mean length of genes (CDS+introns) (bp)	12,898	*11,961	3,788	6,967	6,507
Mean length of transcripts (CDS) (bp)	*2,377	*2,068	1,041	1,766	1,736
Gene models supported by transcriptome (%)	76.30	77.20	72.82	67.02	64.40
Gene Content (total gene length/ total assembled bases x 100) (%)	*78.37	*68.12	14.93	24.36	16.51
G+C content of CDS (%)	*57.67	*50.80	*52.69	56.70	54.95
**Exons**					
Number of exons per gene	21.8	*19.6	4.1	10	8.7
Average length (bp)	109.5	*100.8	256	175.9	199.5
Total length (Mb)	117.3	*83.0	38.4	63.4	46.2
**Introns**					
Number of genes with introns (%)	98.2	*94.7	64.1	92.9	94
Average length (bp)	504.7	*500.4	893	575.1	619.4
Total length (Mb)	516.1	*332.2	101.2	186.8	126.9
**Intergenic regions**					
Average length (bp)	3,633	*1,993	17,888	10,627	23,042

*: values calculated in this study

Supplementary Table 9. Percentage of donor splice sites in *Symbiodinium* genomes.

***Symbiodinium* genome**	**Percentage of 5′-donor splice sites**			**G following AG acceptor sites**	**References and/or remarks**
	**GA** (non-canonical)	**GC** (canonical)	**GT** (non-canonical)		
S. microadriaticum (clade A)	21.9	52.1	26.0	96.2	Aranda et al. 2016
S. minutum (clade B)	15.3	35.9	47.0	93.7	Shoguchi et al. 2013; numbers from Aranda et al. 2016
S. kawagutii (clade F)	2.3	23.2	65.6	86.4	Lin et al. 2015; numbers from Aranda et al. 2016. No splice sites were specified in gene prediction in Lin et al. 2015.
S. goreaui (clade C)	20.6	43.0	36.3	95.5	**This study**
S. kawagutii (clade F)	19.4	36.3	44.3	94.4	**This study**

Supplementary Table 10. Syntenic collinear blocks between each possible pair of four *Symbiodinium* genomes. Each genome is identified by the clade: *S. microadriaticum* (A), *S. minutum* (B), *S. goreaui* (C) and *S. kawagutii* (F).

Number of genes in a block	Number of identified collinear blocks in each genome-pair						Number of genes implicated in collinear blocks in each genome-pair					
	A-B	A-C	A-F	B-C	B-F	C-F	A-B	A-C	A-F	B-C	B-F	C-F
5	1	2	0	4	1	2	5	10	0	20	5	10
6	152	42	52	58	65	99	912	252	312	348	390	594
7	77	24	30	47	36	220	539	168	210	329	252	1540
8	51	11	18	22	26	154	408	88	144	176	208	1232
9	32	4	8	9	17	110	288	36	72	81	153	990
10	23	1	3	7	13	73	230	10	30	70	130	730
11	11	0	2	2	9	64	121	0	22	22	99	704
12	7	2	4	3	2	33	84	24	48	36	24	396
13	7	0	2	1	0	25	91	0	26	13	0	325
14	2	0	1	0	1	30	28	0	14	0	14	420
15	4	0	1	2	1	17	60	0	15	30	15	255
16	1	0	0	0	1	8	16	0	0	0	16	128
17	2	0	0	0	1	11	34	0	0	0	17	187
18	0	0	0	0	0	5	0	0	0	0	0	90
19	0	0	0	0	0	6	0	0	0	0	0	114
20	0	0	0	0	0	4	0	0	0	0	0	80
21	0	0	0	0	0	3	0	0	0	0	0	63
22	0	0	0	0	0	3	0	0	0	0	0	66
23	0	0	0	0	0	1	0	0	0	0	0	23
24	0	0	0	0	0	2	0	0	0	0	0	48
25	0	0	0	0	0	2	0	0	0	0	0	50
26	0	0	0	0	0	2	0	0	0	0	0	52
27	0	0	0	0	0	4	0	0	0	0	0	108
28	0	0	0	0	0	2	0	0	0	0	0	56
29	0	0	0	0	0	0	0	0	0	0	0	0
30	0	0	0	0	0	2	0	0	0	0	0	60
31	0	0	0	0	0	1	0	0	0	0	0	31
32	0	0	0	0	0	1	0	0	0	0	0	32
33	0	0	0	0	0	1	0	0	0	0	0	33
34	0	0	0	0	0	0	0	0	0	0	0	0
35	0	0	0	0	0	0	0	0	0	0	0	0
36	0	0	0	0	0	1	0	0	0	0	0	36
37	0	0	0	0	0	0	0	0	0	0	0	0
38	0	0	0	0	0	0	0	0	0	0	0	0
39	0	0	0	0	0	1	0	0	0	0	0	39
40	0	0	0	0	0	0	0	0	0	0	0	0
53	0	0	0	0	0	1	0	0	0	0	0	53
76	0	0	0	0	0	1	0	0	0	0	0	76
**TOTAL**	**370**	**86**	**121**	**155**	**173**	**889**	**2816**	**588**	**893**	**1125**	**1323**	**8621**

Supplementary Table 11. Statistics of annotated gene models in *S. goreaui* and *S. kawagutii*.

	S. goreaui		***S. kawagutii***	
	number	%	number	%
Total number of gene models	35,913	100.00	26,609	100.00
Number of gene models with UniProt hits	31,646	88.12	21,947	82.48
Number of gene models with Swiss-Prot top hits	17,420	48.51	9,966	37.45
Number of gene models with TrEMBL top hits	14,226	39.61	11,981	45.03
Number of gene models with GO terms associated with Swiss-Prot or TrEMBL hits	29,198	81.30	20,153	75.74
Number of gene models with association with KEGG orthologs	11,604	32.31	6,162	23.16
Number of gene models with annotated Pfam domains	19,718	54.90	11,628	43.70

Supplementary Table 12. List of top ten most abundant protein domains in *Symbiodinium*.

Pfam ID	Domain	Count
***Symbiodinium goreaui* (Clade C)**		
PF00069	Protein kinase domain	494
PF00005	ABC transporter	441
PF07727	Reverse transcriptase (RNA-dependent DNA polymerase)	344
PF00520	Ion transport protein	314
PF12796	Ankyrin repeats (3 copies)	292
PF00072	Response regulator receiver domain	226
PF02518	Histidine kinase-, DNA gyrase B-, and HSP90-like ATPase	220
PF00884	Sulfatase	218
PF07690	Major Facilitator Superfamily	198
PF00145	C-5 cytosine-specific DNA methylase	176
***Symbiodinium kawagutii* (Clade F)**		
PF07727	Reverse transcriptase (RNA-dependent DNA polymerase)	538
PF00145	C-5 cytosine-specific DNA methylase	445
PF00069	Protein kinase domain	374
PF12796	Ankyrin repeats (3 copies)	302
PF00078	Reverse transcriptase (RNA-dependent DNA polymerase)	260
PF00520	Ion transport protein	246
PF00665	Integrase core domain	177
PF00005	ABC transporter	149
PF00075	RNase H	124
PF00226	DnaJ domain	122
***Symbiodinium microadriaticum* (Clade A)**		
PF07727	Reverse transcriptase (RNA-dependent DNA polymerase)	1347
PF12796	Ankyrin repeats (3 copies)	994
PF00078	Reverse transcriptase (RNA-dependent DNA polymerase)	797
PF00069	Protein kinase domain	745
PF00520	Ion transport protein	640
PF00665	Integrase core domain	368
PF13499	EF-hand domain pair	304
PF00226	DnaJ domain	239
PF13637	Ankyrin repeats (many copies)	230
PF13812	Pentatricopeptide repeat domain	217
***Symbiodinium minutum* (Clade B)**		
PF07727	Reverse transcriptase (RNA-dependent DNA polymerase)	772
PF00069	Protein kinase domain	756
PF12796	Ankyrin repeats (3 copies)	679
PF00520	Ion transport protein	668
PF13499	EF-hand domain pair	386
PF00226	DnaJ domain	285
PF13041	PPR repeat family	216
PF13812	Pentatricopeptide repeat domain	215
PF00076	RNA recognition motif. (a.k.a. RRM; RBD; or RNP domain)	211
PF00665	Integrase core domain	211

Supplementary Table 13. Reference proteins from 31 eukaryote species used in the Pfam domain enrichment analysis.

Phylum/ Group	Subgroup	Species	Data source
Alveolata	Apicomplexa	*Plasmodium falciparum*	ftp://ftp.ensemblgenomes.org/pub/protists/release-32/fasta/plasmodium_falciparum/pep/Plasmodium_falciparum.ASM276v1.pep.all.fa.gz
Alveolata	Apicomplexa	*Toxoplasma gondii*	ftp://ftp.ensemblgenomes.org/pub/protists/release-32/fasta/toxoplasma_gondii/pep/Toxoplasma_gondii.ToxoDB-7.1.pep.all.fa.gz
Alveolata	Ciliate	*Paramecium biaurelia*	http://paramecium.cgm.cnrs-gif.fr/download/species/pbiaurelia/biaurelia_V1-4_annotation_v1.protein.fa
Alveolata	Ciliate	*Paramecium caudatum*	http://paramecium.cgm.cnrs-gif.fr/download/species/pcaudatum/caudatum_43c3d_annotation_v1.protein.fa
Alveolata	Ciliate	*Paramecium sexaurelia*	http://paramecium.cgm.cnrs-gif.fr/download/species/psexaurelia/sexaurelia_AZ8-4_annotation_v1.protein.fa
Alveolata	Ciliate	*Paramecium tetraurelia*	ftp://ftp.ensemblgenomes.org/pub/protists/release-32/fasta/paramecium_tetraurelia/pep/Paramecium_tetraurelia.GCA_000165425.1.pep.all.fa.gz
Alveolata	Dinoflagellate*	*Alexandrium tamarense* CCMP1771; MMETSP0382	http://datacommons.cyverse.org/browse/iplant/home/shared/imicrobe/projects/
Alveolata	Dinoflagellate*	*Amphidinium carterae* CCMP1314; MMETSP0398/MMETSP0399/ MMETSP0258/MMETSP0259	http://datacommons.cyverse.org/browse/iplant/home/shared/imicrobe/projects/
Alveolata	Dinoflagellate*	*Gambierdiscus australes* CAWD149; MMETSP0766	http://datacommons.cyverse.org/browse/iplant/home/shared/imicrobe/projects/
Alveolata	Dinoflagellate*	*Heterocapsa triquetra* CCMP448; MMETSP0448	http://datacommons.cyverse.org/browse/iplant/home/shared/imicrobe/projects/
Alveolata	Dinoflagellate*	*Karenia brevis* CCMP2229; MMETSP0029	http://datacommons.cyverse.org/browse/iplant/home/shared/imicrobe/projects/
Alveolata	Dinoflagellate*	*Karlodinium micrum* CCMP2283; MMETSP1015	http://datacommons.cyverse.org/browse/iplant/home/shared/imicrobe/projects/
Alveolata	Dinoflagellate*	*Lingulodinium polyedra* CCMP1738; MMETSP1033	http://datacommons.cyverse.org/browse/iplant/home/shared/imicrobe/projects/
Alveolata	Dinoflagellate*	*Polarella glacialis* CCMP1383; MMETSP0227; CCMP2088; MMETSP1440	http://datacommons.cyverse.org/browse/iplant/home/shared/imicrobe/projects/
Alveolata	Dinoflagellate*	*Prorocentrum minimum* CCMP1329; MMETSP0053	http://datacommons.cyverse.org/browse/iplant/home/shared/imicrobe/projects/
Alveolata	Dinoflagellate*	*Scrippsiella hangoei* SHTV5; MMETSP0359/MMETSP0360	http://datacommons.cyverse.org/browse/iplant/home/shared/imicrobe/projects/
Alveolata	Dinoflagellate*	*Symbiodinium goreaui*	**This study**
Alveolata	Dinoflagellate*	*Symbiodinium kawagutii*	**This study**
Alveolata	Dinoflagellate*	*Symbiodinium microadriaticum*	http://smic.reefgenomics.org/download/Smic.genome.annotation.pep.longest.fa.gz
Alveolata	Dinoflagellate*	*Symbiodinium minutum*	http://marinegenomics.oist.jp/symb/download/symbB.v1.2.augustus.prot.fa.gz
Alveolata	Perkinsea*	*Perkinsus marinus*	http://mirrors.vbi.vt.edu/mirrors/ftp.ncbi.nih.gov/genomes/refseq/protozoa/Perkinsus_marinus/representative/GCF_000006405.1_JCVI_PMG_1.0/GCF_000006405.1_JCVI_PMG_1.0_protein.faa.gz
Stramenopiles	Diatom	*Phaeodactylum tricornutum*	ftp://ftp.ensemblgenomes.org/pub/protists/release-32/fasta/phaeodactylum_tricornutum/pep/Phaeodactylum_tricornutum.ASM15095v2.pep.all.fa.gz
Stramenopiles	Diatom	*Thalassiosira pseudonana*	ftp://ftp.ensemblgenomes.org/pub/protists/release-32/fasta/thalassiosira_pseudonana/pep/Thalassiosira_pseudonana.ASM14940v2.pep.all.fa.gz
Archaeplastida	Angiosperm	*Arabidopsis thaliana*	ftp://ftp.arabidopsis.org/home/tair/Proteins/TAIR10_protein_lists/TAIR10_pep_20101214
Archaeplastida	Green algae	*Chlamydomonas reinhardtii*	ftp://ftp.ensemblgenomes.org/pub/plants/release-32/fasta/chlamydomonas_reinhardtii/pep/Chlamydomonas_reinhardtii.v3.1.pep.all.fa.gz
Archaeplastida	Green algae	*Micromonas commoda* RCC299	http://genome.jgi.doe.gov/pages/dynamicOrganismDownload.jsf?organism=Phytozome
Archaeplastida	Green algae	*Micromonas pusilla* CCMP1545	ftp://ftp.ncbi.nlm.nih.gov/genomes/all/GCF/000/151/265/GCF_000151265.2_Micromonas_pusilla_CCMP1545_v2.0/GCF_000151265.2_Micromonas_pusilla_CCMP1545_v2.0_protein.faa.gz
Archaeplastida	Red algae	*Chondrus crispus*	ftp://ftp.ensemblgenomes.org/pub/plants/release-32/fasta/plants_rhodophyta1_collection/chondrus_crispus/pep/Chondrus_crispus.ASM35022v2.pep.all.fa.gz
Archaeplastida	Red algae	*Cyanidioschyzon merolae*	ftp://ftp.ensemblgenomes.org/pub/plants/release-32/fasta/cyanidioschyzon_merolae/pep/Cyanidioschyzon_merolae.ASM9120v1.pep.all.fa.gz
Archaeplastida	Red algae	*Galdieria sulphuraria*	ftp://ftp.ensemblgenomes.org/pub/plants/release-32/fasta/plants_rhodophyta1_collection/galdieria_sulphuraria/pep/Galdieria_sulphuraria.ASM34128v1.pep.all.fa.gz
Archaeplastida	Red algae	*Porphyridium purpureum*	http://cyanophora.rutgers.edu/porphyridium/Porphyridium_genemodels_UPDATED.fasta

*: The 14 dinoflagellates and 1 Perkinsea species constitute the 15-taxon set.

Supplementary Table 14. Homologous protein sets used in this study.

	31-taxon set	15-taxon set
Number of proteins	1,136,347	880,969
Number of homologous sets*	56,530	44,282
Number of singletons	310,288	266,335
Number of homologous proteins in a set	826,059	614,634
Percentage of proteins in homologous sets	72.20	69.80
Number of orthogroups containing only proteins from *Symbiodinium*^	2,776	3,180
Number of *Symbiodinium* singletons^	21,812	24,508
Mean size of homologous protein sets	14.60	13.90

*: a set here is referred to as an "orthogroup" in OrthoFinder (Emms and Kelly, 2015); each "orthogroup" consists of homologous proteins including orthologs and paralogs.

^: all proteins in these two sets are considered *Symbiodinium*-specific.

Supplementary Table 15. *Symbiodinium* genes with putative functions relevant to mycosporine-like amino acid biosynthesis.

Query group	Query - source of organism	Query - putative function	Gene name	UniProt Identifer	Query protein description	Query len.	***S. microadriaticum* (clade A)**			***S. minutum* (clade B)**			***S. goreaui* (clade C)**			***S. kawagutii* (clade F)**		
							Hit	E-value	Aln len.	Hit	E-value	Aln len.	Hit	E-value	Aln len.	Hit	E-value	Aln len.
Actinobacteria	Actinosynnema mirum DSM 43827	ATP-grasp	ACU38111.1	C6WIM1_ACTMD	D-alanine--D-alanine ligase	339	Smic31738	7.00E-75	335	NA	NA	NA	SymbC1.scaffold2594.3	8.00E-25	185	SymbF.scaffold249.18	3.00E-10	198
Actinobacteria	Actinosynnema mirum DSM 43827	ATP-grasp	ACU38112.1	C6WIM2_ACTMD	Uncharacterized protein	429	Smic19143	6.00E-35	351	NA	NA	NA	NA	NA	NA	NA	NA	NA
Actinobacteria	Pseudonocardia sp. P1	ATP-grasp	WP_010243315.1	WP_010243315.1 (NCBI)	D-alanine--D-alanine ligase	346	Smic31738	3.00E-67	332	NA	NA	NA	SymbC1.scaffold2594.3	2.00E-24	183	SymbF.scaffold37.151	3.00E-09	129
Actinobacteria	Pseudonocardia sp. P1	ATP-grasp	WP_010243317.1	WP_010243317.1 (NCBI)	ATP-grasp domain-containing protein	411	Smic19143	1.00E-33	308	NA	NA	NA	NA	NA	NA	NA	NA	NA
Cnidaria	Nematostella vectensis	ATP-grasp	EDO41319	A7S4P1_NEMVE	ATP-grasp domain-containing protein	594	Smic19143	3.00E-19	307	NA	NA	NA	NA	NA	NA	NA	NA	NA
Cyanobacteria	Anabaena variabilis ATCC 29413	ATP-grasp	ava:Ava_3856	Q3M6C5_ANAVT	ATP-grasp enzyme-like protein	458	Smic19143	3.00E-43	350	NA	NA	NA	NA	NA	NA	NA	NA	NA
Cyanobacteria	Aphanothece halophytica	ATP-grasp	saltStress:BAO51916.1	W8VR81_APHHA	D-alanine--D-alanine ligase	339	Smic31738	2.00E-59	350	NA	NA	NA	SymbC1.scaffold2594.3	4.00E-27	159	SymbF.scaffold249.18	2.00E-10	203
Cyanobacteria	Aphanothece halophytica	ATP-grasp	saltStress:BAO51915.1	W8VTF0_APHHA	C-N ligase	444	Smic19143	1.00E-43	332	NA	NA	NA	NA	NA	NA	NA	NA	NA
Cyanobacteria	Nostoc punctiforme ATCC 29133	ATP-grasp	npu:Npun_F5597	B2J6X6_NOSP7	D-alanine--D-alanine ligase	348	Smic31738	3.00E-78	335	NA	NA	NA	SymbC1.scaffold2594.3	5.00E-29	185	SymbF.scaffold249.18	6.00E-11	203
Cyanobacteria	Nostoc punctiforme ATCC 29133	ATP-grasp	npu:Npun_R5598	B2J6X7_NOSP7	Uncharacterized protein	461	Smic19143	1.00E-39	369	NA	NA	NA	NA	NA	NA	NA	NA	NA
Cyanobacteria	Scytonema cf crispum UCFS10	ATP-grasp	ANY58985.1	A0A1B2CWF7_9CYAN	ATP-grasp domain-containing protein	463	Smic19143	5.00E-40	392	NA	NA	NA	NA	NA	NA	NA	NA	NA
Cyanobacteria	Scytonema cf crispum UCFS10	ATP-grasp	ANY58992.1	A0A1B2CWG9_9CYAN	ATP-grasp domain-containing protein	463	Smic19143	5.00E-40	392	NA	NA	NA	NA	NA	NA	NA	NA	NA
Cyanobacteria	Scytonema cf crispum UCFS15	ATP-grasp	ACLA_055830	A1C9L1_ASPCL	Uncharacterized protein	496	Smic19143	2.00E-25	391	NA	NA	NA	NA	NA	NA	NA	NA	NA
Actinobacteria	Actinosynnema mirum DSM 43827	DHQS	ACU38114.1	C6WIM4_ACTMD	3-dehydroquinate synthase	406	Smic43919	4.00E-64	366	symbB.v1.2.028953.t1	3.00E-28	244	SymbC1.scaffold2.2020	6.00E-46	346	SymbF.scaffold11.23	5.00E-28	343
Actinobacteria	Pseudonocardia sp. P1	DHQS	WP_010243321.1	WP_010243321.1 (NCBI)	3-dehydroquinate synthase	409	Smic43919	5.00E-69	369	symbB.v1.2.028953.t1	3.00E-29	244	SymbC1.scaffold21.53	8.00E-41	341	SymbF.scaffold11.23	3.00E-33	335
Actinobacteria	Streptomyces hygroscopicus subsp. jinggangensis 5008	DHQS	4P53:A|PDBID|CHAIN|SEQUENCE	VALA_STRHJ	2-epi-5-epi-valiolone synthase	420	Smic43919	7.00E-58	356	symbB.v1.2.028953.t1	6.00E-34	274	SymbC1.scaffold2.2020	8.00E-64	380	SymbF.scaffold11.23	3.00E-31	355
Cnidaria	Nematostella vectensis	DHQS	EDO40476	A7S759_NEMVE	DHQ synthase	352	Smic43919	1.00E-172	351	symbB.v1.2.028953.t1	4.00E-24	253	SymbC1.scaffold2.2020	3.00E-26	345	SymbF.scaffold11.23	4.00E-21	333
Cyanobacteria	Anabaena variabilis ATCC 29413	DHQS	ava:Ava_3858	DDGS_ANAVT	Demethyl-4-deoxygadusol synthase	410	Smic43919	6.00E-76	372	symbB.v1.2.028953.t1	7.00E-27	242	SymbC1.scaffold2.2020	8.00E-42	349	SymbF.scaffold11.23	3.00E-23	324
Cyanobacteria	Aphanothece halophytica	DHQS	saltStress:BAO51913.1	W8VKF5_APHHA	Demethyl 4-deoxygadusol synthase	587	Smic35309	4.00E-173	569	symbB.v1.2.028953.t1	3.00E-21	248	SymbC1.scaffold8151.2	2.00E-36	283	SymbF.scaffold11.23	4.00E-15	334
Cyanobacteria	Nostoc punctiforme ATCC 29133	DHQS	npu:Npun_R5600	DDGS_NOSP7	Demethyl-4-deoxygadusol synthase	410	Smic2116	2.00E-71	370	symbB.v1.2.028953.t1	8.00E-25	242	SymbC1.scaffold2.2020	4.00E-48	332	SymbF.scaffold11.23	1.00E-26	322
Cyanobacteria	Scytonema cf crispum UCFS10	DHQS	ANY58986.1	A0A1B2CWG1_9CYAN	3-dehydroquinate synthase	409	Smic43919	1.00E-72	382	symbB.v1.2.028953.t1	2.00E-26	246	SymbC1.scaffold2.2020	2.00E-50	335	SymbF.scaffold11.23	2.00E-31	340
Cyanobacteria	Scytonema cf crispum UCFS15	DHQS	ANY58993.1	A0A1B2CWG5_9CYAN	3-dehydroquinate synthase	409	Smic43919	1.00E-72	382	symbB.v1.2.028953.t1	2.00E-26	246	SymbC1.scaffold2.2020	2.00E-50	335	SymbF.scaffold11.23	2.00E-31	340
Fungi	Aspergillus clavatus NRRL 1	DHQS	ACLA_055850	A1C9L3_ASPCL	3-dehydroquinate synthase, putative	459	Smic2116	3.00E-72	371	symbB.v1.2.028953.t1	7.00E-28	265	SymbC1.scaffold2.2020	2.00E-34	351	SymbF.scaffold11.23	3.00E-25	328
Fungi	Aspergillus nidulans FGSC A4	DHQS	tr|Q5AZ77|Q5AZ77_EMENI	Q5AZ77_EMENI	3-dehydroquinate synthase	483	Smic43919	1.00E-69	368	symbB.v1.2.028953.t1	6.00E-25	265	SymbC1.scaffold2.2020	3.00E-34	351	SymbF.scaffold11.23	2.00E-24	327
Metazoa	Danio rerio	DHQS	tr|E7EXW6|E7EXW6_DANRE	E7EXW6_DANRE	2-epi-5-epi-valiolone synthase	470	Smic2116	1.00E-49	413	symbB.v1.2.028953.t1	6.00E-27	259	SymbC1.scaffold2.2020	2.00E-48	368	SymbF.scaffold11.23	9.00E-24	339
Metazoa	Danio rerio	DHQS	tr|Q5BLE6|Q5BLE6_DANRE	Q5BLE6_DANRE	Zgc:113054	551	Smic2116	3.00E-45	268	symbB.v1.2.020911.t1	3.00E-29	264	SymbC1.scaffold2.1851	4.00E-38	248	SymbF.scaffold11.10	7.00E-36	243
Dinoflagellata	Heterocapsa triquetra	DHQS/O-MT	ABF61766	Q15BR2_HETTR	Chloroplast 3-dehydroquinate synthase/O-methyltransferase fusion	951	Smic2116	0.00	943	symbB.v1.2.028953.t1	2.00E-19	255	SymbC1.scaffold8151.2	2.00E-90	269	SymbF.scaffold313.28	8.00E-11	140
Cyanobacteria	Anabaena variabilis ATCC 29413	NRPS	ava:Ava_3855	Q3M6C6_ANAVT	Amino acid adenylation	888	Smic3690	2.00E-101	616	symbB.v1.2.012436.t1	8.00E-78	474	SymbC1.scaffold70.75	0.00	720	SymbF.scaffold1354.11	4.00E-68	481
Cyanobacteria	Scytonema cf crispum UCFS10	NRPS	ANY58984.1	A0A1B2CWG3_9CYAN	Non-ribosomal synthetase	1328	Smic3690	7.00E-104	808	symbB.v1.2.012436.t1	6.00E-90	982	SymbC1.scaffold70.75	0.00	1169	SymbF.scaffold213.33	3.00E-63	635
Cyanobacteria	Scytonema cf crispum UCFS15	NRPS	ANY58991.1	A0A1B2CWH8_9CYAN	Non-ribosomal synthetase	1328	Smic3690	7.00E-104	808	symbB.v1.2.012436.t1	6.00E-90	982	SymbC1.scaffold70.75	0.00	1169	SymbF.scaffold213.33	3.00E-63	635
Actinobacteria	Actinosynnema mirum DSM 43827	O-MT	ACU38113.1	C6WIM3_ACTMD	O-methyltransferase family 3	284	Smic1333	2.00E-38	230	symbB.v1.2.006248.t1	2.00E-26	191	SymbC1.scaffold2377.8	2.00E-28	192	SymbF.scaffold308.24	9.00E-13	113
Actinobacteria	Pseudonocardia sp. P1	O-MT	WP_010243319.1	WP_010243319.1 (NCBI)	SAM-dependent methyltransferase	250	Smic2116	2.00E-42	257	symbB.v1.2.006248.t1	7.00E-19	241	SymbC1.scaffold25.7	8.00E-23	175	SymbF.scaffold313.28	2.00E-10	160
Cnidaria	Nematostella vectensis	O-MT	EDO40475	A7S758_NEMVE	O-methyltransferase activity	174	Smic1333	2.00E-27	179	symbB.v1.2.006248.t1	6.00E-12	185	SymbC1.scaffold25.7	1.00E-21	168	SymbF.scaffold308.24	6.00E-07	109

Query group	Query - source of organism	Query - putative function	Gene name	UniProt Identifer	Query protein description	Query len.	***S. microadriaticum* (clade A)**			***S. minutum* (clade B)**			***S. goreaui* (clade C)**			***S. kawagutii* (clade F)**		
							Hit	E-value	Aln len.	Hit	E-value	Aln len.	Hit	E-value	Aln len.	Hit	E-value	Aln len.
Cyanobacteria	Anabaena variabilis ATCC 29413	O-MT	ava:Ava_3857	Q3M6C4_ANAVT	O-methyltransferase family 3	279	Smic2116	5.00E-45	238	symbB.v1.2.006248.t1	5.00E-25	194	SymbC1.scaffold2377.8	5.00E-33	195	SymbF.scaffold308.24	1.00E-10	116
Cyanobacteria	Aphanothece halophytica	O-MT	saltStress:BAO51914.1	W8VY25_APHHA	O-methyltransferase	279	Smic2116	2.00E-49	273	symbB.v1.2.018440.t1	6.00E-20	216	SymbC1.scaffold25.7	4.00E-26	174	SymbF.scaffold308.24	2.00E-13	122
Cyanobacteria	Nostoc punctiforme ATCC 29133	O-MT	npu:Npun_R5599	B2J6X8_NOSP7	O-methyltransferase family 3	277	Smic1333	1.00E-45	253	symbB.v1.2.018440.t1	2.00E-24	205	SymbC1.scaffold2377.8	8.00E-28	191	SymbF.scaffold308.24	4.00E-11	124
Cyanobacteria	Scytonema cf crispum UCFS15	O-MT	ANY58987.1	A0A1B2CWH0_9CYAN	O-methyltransferase	296	Smic2116	2.00E-47	225	symbB.v1.2.018440.t1	1.00E-28	200	SymbC1.scaffold2377.8	1.00E-28	191	SymbF.scaffold308.24	1.00E-12	124
Cyanobacteria	Scytonema cf crispum UCFS15	O-MT	ANY58994.1	A0A1B2CWH5_9CYAN	O-methyltransferase	296	Smic2116	2.00E-47	225	symbB.v1.2.018440.t1	1.00E-28	200	SymbC1.scaffold2377.8	1.00E-28	191	SymbF.scaffold308.24	1.00E-12	124
Fungi	Aspergillus clavatus NRRL 1	O-MT	ACLA_055840	A1C9L2_ASPCL	O-methyltransferase putative	286	Smic31139	9.00E-24	194	symbB.v1.2.014617.t2	5.00E-26	201	SymbC1.scaffold2416.1	3.00E-23	179	SymbF.scaffold593.9	1.00E-23	205
Fungi	Aspergillus nidulans FGSC A4	O-MT/ATP-grasp	tr|Q5AZ78|Q5AZ78_EMENI	Q5AZ78_EMENI	Uncharacterized protein	816	Smic19143	2.00E-26	402	symbB.v1.2.014617.t2	3.00E-26	240	SymbC1.scaffold5773.1	9.00E-22	183	SymbF.scaffold593.9	1.00E-23	205
Cyanobacteria	Scytonema cf crispum UCFS10	Short-chain dehydrogenase	ANY58988.1	A0A1B2CWF8_9CYAN	Short-chain dehydrogenase	293	Smic1413	5.00E-27	236	symbB.v1.2.020911.t1	3.00E-24	258	SymbC1.scaffold2890.25	1.00E-34	252	SymbF.scaffold270.25	1.00E-24	233
Cyanobacteria	Scytonema cf crispum UCFS10	Short-chain dehydrogenase	ANY58995.1	A0A1B2CWH2_9CYAN	short-chain dehydrogenase	293	Smic1413	5.00E-27	236	symbB.v1.2.020911.t1	3.00E-24	258	SymbC1.scaffold2890.25	1.00E-34	252	SymbF.scaffold270.25	1.00E-24	233

Supplementary Table 16. BLASTP hits ($E < 10^{-5}$) in *Symbiodinium* for each of the 18 *Nostoc punctiforme* proteins involved in scytonemin biosynthesis.

Protein	***S. microadriaticum* (A)**		***S. minutum* (B)**		***S. goreaui* (C)**		***S. kawagutii* (F)**	
	Hit(s)	E-value	Hit(s)	E-value	Hit(s)	E-value	Hit(s)	E-value
UniProt ID: B2IXH5<br>Gene: *scyA*<br>Gene ID in *N. punctiforme*: Npun_R1276	Smic28555<br>Smic21519<br>Smic26902	5.00E-14<br>3.00E-11<br>6.00E-11	symbB.v1.2.035008.t1<br>symbB.v1.2.032800.t1<br>symbB.v1.2.034800.t2<br>symbB.v1.2.034800.t1	5.00E-19<br>8.00E-14<br>1.00E-10<br>2.00E-10	SymbC1.scaffold2.2026<br>SymbC1.scaffold1.2344<br>SymbC1.scaffold4.363<br>SymbC1.scaffold4.284<br>SymbC1.scaffold9.174<br>SymbC1.scaffold5.1134<br>SymbC1.scaffold25.119<br>SymbC1.scaffold1.1299<br>SymbC1.scaffold5.414<br>SymbC1.scaffold540.25<br>SymbC1.scaffold2.1375<br>SymbC1.scaffold4.1019<br>SymbC1.scaffold1.1273<br>SymbC1.scaffold4.971<br>SymbC1.scaffold2.950<br>SymbC1.scaffold109.61<br>SymbC1.scaffold35850.1<br>SymbC1.scaffold241.78<br>SymbC1.scaffold15.162<br>SymbC1.scaffold2.1994<br>SymbC1.scaffold5156.2<br>SymbC1.scaffold5001.1<br>SymbC1.scaffold4102.1<br>SymbC1.scaffold3142.3<br>SymbC1.scaffold1672.2<br>SymbC1.scaffold21415.1<br>SymbC1.scaffold5131.1<br>SymbC1.scaffold241.24	1.00E-40<br>5.00E-32<br>2.00E-27<br>4.00E-27<br>6.00E-27<br>2.00E-25<br>1.00E-24<br>1.00E-23<br>1.00E-22<br>4.00E-22<br>1.00E-20<br>2.00E-20<br>6.00E-20<br>8.00E-20<br>2.00E-19<br>3.00E-19<br>1.00E-18<br>2.00E-18<br>4.00E-18<br>8.00E-17<br>3.00E-15<br>1.00E-14<br>1.00E-12<br>7.00E-08<br>9.00E-08<br>7.00E-07<br>7.00E-07<br>7.00E-06	SymbF.scaffold35.95<br>SymbF.scaffold3271.1<br>SymbF.scaffold520.14<br>SymbF.scaffold1982.4	3.00E-28<br>3.00E-20<br>7.00E-16<br>4.00E-08
UniProt ID: B2IXH4<br>Gene: *scyB*<br>Gene ID in *N. punctiforme*: Npun_R1275	No hits	N/A	No hits	N/A	SymbC1.scaffold1093.35<br>SymbC1.scaffold2.1837<br>SymbC1.scaffold14551.5<br>SymbC1.scaffold32.93<br>SymbC1.scaffold37069.1	6.00E-83<br>3.00E-22<br>8.00E-18<br>2.00E-14<br>1.00E-11	SymbF.scaffold8.273<br>SymbF.scaffold3240.3	8.00E-86<br>5.00E-06
UniProt ID: B2IXH3<br>Gene: *scyC*<br>Gene ID in *N. punctiforme*: Npun_R1274	No hits	N/A	No hits	N/A	No hits	N/A	No hits	N/A
UniProt ID: B2IXH2<br>Gene: *scyD*<br>Gene ID in *N. punctiforme*: Npun_R1273	No hits	N/A	No hits	N/A	No hits	N/A	No hits	N/A
UniProt ID: B2IXH1<br>Gene: *scyE*<br>Gene ID in *N. punctiforme*: Npun_R1272	No hits	N/A	No hits	N/A	No hits	N/A	No hits	N/A
UniProt ID: B2IXH0<br>Gene: *scyF*<br>Gene ID in *N. punctiforme*: Npun_R1271	Smic37965<br>Smic11872<br>Smic722<br>Smic37012	4.00E-16<br>6.00E-12<br>1.00E-11<br>2.00E-06	symbB.v1.2.008932.t1<br>symbB.v1.2.008932.t1<br>symbB.v1.2.000102.t1<br>symbB.v1.2.034012.t1<br>symbB.v1.2.040186.t1	3.00E-14<br>9.00E-10<br>2.00E-13<br>3.00E-08<br>1.00E-07	SymbC1.scaffold1093.35<br>SymbC1.scaffold2.1837<br>SymbC1.scaffold14551.5<br>SymbC1.scaffold32.93<br>SymbC1.scaffold37069.1<br>SymbC1.scaffold4.1475<br>SymbC1.scaffold8194.1	6.00E-83<br>3.00E-22<br>8.00E-18<br>2.00E-14<br>1.00E-11<br>1.00E-07<br>6.00E-07	SymbF.scaffold74.4<br>SymbF.scaffold27.38	4.00E-07<br>6.00E-06

Protein	***S. microadriaticum* (A)**		***S. minutum* (B)**		***S. goreaui* (C)**		***S. kawagutii* (F)**	
	Hit(s)	E-value	Hit(s)	E-value	Hit(s)	E-value	Hit(s)	E-value
UniProt ID: B2IXG9<br>Gene: Glycosyl-transferase<br>Gene ID in *N. punctiforme*: Npun_R1270	Smic44385<br>Smic5216	9.00E-12<br>8.00E-06	symbB.v1.2.002866.t1<br>symbB.v1.2.002866.t2<br>symbB.v1.2.026225.t1	2.00E-09<br>2.00E-09<br>3.00E-07	SymbC1.scaffold410.29<br>SymbC1.scaffold2.386<br>SymbC1.scaffold9.56<br>SymbC1.scaffold17.9<br>SymbC1.scaffold5.1082<br>SymbC1.scaffold16.67<br>SymbC1.scaffold2.875<br>SymbC1.scaffold80.139<br>SymbC1.scaffold2.657<br>SymbC1.scaffold1.992<br>SymbC1.scaffold2.660<br>SymbC1.scaffold1401.15<br>SymbC1.scaffold5.635	1.00E-42<br>4.00E-39<br>1.00E-26<br>1.00E-24<br>1.00E-22<br>1.00E-17<br>8.00E-09<br>1.00E-08<br>4.00E-08<br>9.00E-08<br>2.00E-07<br>2.00E-06<br>5.00E-06	SymbF.scaffold2862.5	4.00E-07
UniProt ID: B2IXG8<br>Gene: *tyrA*<br>Gene ID in *N. punctiforme*: Npun_R1269	No hits	N/A	No hits	N/A	No hits	N/A	No hits	N/A
UniProt ID: B2IXG7<br>Gene: *dsbA*<br>Gene ID in *N. punctiforme*: Npun_R1268	Smic39492 (recovered at 1e-4)	1.01E-04	symbB.v1.2.029824.t1	1.00E-33	SymbC1.scaffold216.15<br>SymbC1.scaffold5154.6<br>SymbC1.scaffold19.147	1.00E-23<br>9.00E-12<br>5.00E-09	SymbF.scaffold2870.9	1.00E-12
UniProt ID: B2IXG6<br>Gene: *aroB (DHQS)*<br>Gene ID in *N. punctiforme*: Npun_R1267	Smic42001<br>Smic2116<br>Smic43919<br>Smic35309<br>Smic35461<br>Smic12094	3.00E-32<br>1.00E-21<br>1.00E-21<br>5.00E-21<br>6.00E-16<br>5.00E-13	symbB.v1.2.028953.t1	0.00	SymbC1.scaffold11.271<br>SymbC1.scaffold4.799<br>SymbC1.scaffold9.107<br>SymbC1.scaffold2.2020<br>SymbC1.scaffold21.53<br>SymbC1.scaffold7772.3<br>SymbC1.scaffold685.28<br>SymbC1.scaffold7.65<br>SymbC1.scaffold24348.2<br>SymbC1.scaffold22405.1	8.00E-39<br>2.00E-38<br>1.00E-35<br>4.00E-35<br>5.00E-34<br>9.00E-31<br>3.00E-30<br>6.00E-25<br>1.00E-18<br>7.00E-07	SymbF.scaffold11.23<br>SymbF.scaffold3925.3	2.00E-34<br>2.00E-23
UniProt ID: B2IXG5<br>Gene: *trpE*<br>Gene ID in *N. punctiforme*: Npun_R1266	Smic1909	9.00E-42	symbB.v1.2.004052.t1<br>symbB.v1.2.037466.t1	0.00<br>6.00E-20	SymbC1.scaffold3197.1<br>SymbC1.scaffold22.189<br>SymbC1.scaffold5.278<br>SymbC1.scaffold21.403<br>SymbC1.scaffold13906.1<br>SymbC1.scaffold17352.1<br>SymbC1.scaffold15.265<br>SymbC1.scaffold70.43<br>SymbC1.scaffold2.1336<br>SymbC1.scaffold23.168<br>SymbC1.scaffold70.35<br>SymbC1.scaffold482.52<br>SymbC1.scaffold1.483<br>SymbC1.scaffold80.158<br>SymbC1.scaffold2.1535<br>SymbC1.scaffold9.600<br>SymbC1.scaffold1.688<br>SymbC1.scaffold685.10<br>SymbC1.scaffold3095.7<br>SymbC1.scaffold4.342<br>SymbC1.scaffold7.63	0.00<br>1.00E-53<br>1.00E-53<br>3.00E-49<br>8.00E-39<br>1.00E-33<br>4.00E-33<br>1.00E-30<br>1.00E-27<br>3.00E-23<br>8.00E-21<br>1.00E-19<br>6.00E-16<br>9.00E-16<br>4.00E-11<br>2.00E-09<br>3.00E-08<br>1.00E-07<br>1.00E-07<br>3.00E-06<br>6.00E-06	SymbF.scaffold11.23<br>SymbF.scaffold3925.3	2.00E-34<br>2.00E-23
UniProt ID: B2IXG4<br>Gene: *trpC*<br>Gene ID in *N. punctiforme*: Npun_R1265	Smic7823	3.00E-33	symbB.v1.2.022171.t1	2.00E-34	SymbC1.scaffold17429.1<br>SymbC1.scaffold70.43<br>SymbC1.scaffold80.158<br>SymbC1.scaffold11.333<br>SymbC1.scaffold21.296<br>SymbC1.scaffold17905.1<br>SymbC1.scaffold21766.1<br>SymbC1.scaffold2493.1	3.00E-59<br>1.00E-55<br>1.00E-55<br>5.00E-55<br>3.00E-52<br>4.00E-46<br>6.00E-46<br>6.00E-23	SymbF.scaffold3240.10	1.00E-09
UniProt ID: B2IXG3<br>Gene: *trpA*<br>Gene ID in *N. punctiforme*: Npun_R1264	Smic3643	4.00E-16	symbB.v1.2.000731.t1	2.00E-41	SymbC1.scaffold5.1067<br>SymbC1.scaffold19.272<br>SymbC1.scaffold29630.1<br>SymbC1.scaffold1995.42<br>SymbC1.scaffold4.230<br>SymbC1.scaffold219.88<br>SymbC1.scaffold7.82<br>SymbC1.scaffold27503.1	2.00E-60<br>1.00E-56<br>6.00E-53<br>6.00E-44<br>1.00E-43<br>3.00E-36<br>6.00E-35<br>1.00E-24	SymbF.scaffold3240.10	5.00E-21

Protein	*S. microadriaticum* (A)		*S. minutum* (B)		*S. goreaui* (C)		*S. kawagutii* (F)	
	Hit(s)	E-value	Hit(s)	E-value	Hit(s)	E-value	Hit(s)	E-value
UniProt ID: B2IXG2<br>Gene: *tyrP*<br>Gene ID in *N. punctiforme*: Npun_R1263	Smic7316	2.00E-09	No hits	N/A	No hits	N/A	No hits	N/A
UniProt ID: B2IXG1<br>Gene: *trpB*<br>Gene ID in *N. punctiforme*: Npun_R1262	Smic36430<br>Smic14060	4.00E-37<br>1.00E-30	symbB.v1.2.000731.t1<br>symbB.v1.2.014164.t1	1.00E-154<br>2.00E-32	SymbC1.scaffold1.724<br>SymbC1.scaffold1995.42<br>SymbC1.scaffold4.230<br>SymbC1.scaffold7.82<br>SymbC1.scaffold219.88<br>SymbC1.scaffold5.1067<br>SymbC1.scaffold9.195<br>SymbC1.scaffold29611.1<br>SymbC1.scaffold3205.1<br>SymbC1.scaffold38296.1<br>SymbC1.scaffold2284.2<br>SymbC1.scaffold29630.1<br>SymbC1.scaffold540.17<br>SymbC1.scaffold7.661<br>SymbC1.scaffold2815.2	4.00E-172<br>4.00E-169<br>1.00E-166<br>7.00E-155<br>2.00E-147<br>3.00E-147<br>1.00E-143<br>3.00E-129<br>4.00E-111<br>2.00E-77<br>4.00E-33<br>9.00E-33<br>6.00E-30<br>5.00E-26<br>1.00E-20	SymbF.scaffold11.353<br>SymbF.scaffold939.1<br>SymbF.scaffold15790.1	6.00E-172<br>3.00E-154<br>2.00E-51
UniProt ID: B2IXG0<br>Gene: *trpD*<br>Gene ID in *N. punctiforme*: Npun_R1261	Smic34102	2.00E-47	symbB.v1.2.012378.t1	5.00E-46	SymbC1.scaffold7.227<br>SymbC1.scaffold21.296<br>SymbC1.scaffold80.158<br>SymbC1.scaffold70.43	2.00E-58<br>7.00E-52<br>8.00E-49<br>6.00E-43	SymbF.scaffold3240.10	3.00E-59
UniProt ID: B2IXF9<br>Gene: *aroG*<br>Gene ID in *N. punctiforme*: Npun_R1260	No hits	N/A	No hits	N/A	SymbC1.scaffold11.361<br>SymbC1.scaffold5.979<br>SymbC1.scaffold17.172<br>SymbC1.scaffold2.1765<br>SymbC1.scaffold1.197<br>SymbC1.scaffold8922.3<br>SymbC1.scaffold70.31<br>SymbC1.scaffold18.265<br>SymbC1.scaffold4.307<br>SymbC1.scaffold216.60	3.00E-90<br>3.00E-87<br>3.00E-81<br>2.00E-78<br>2.00E-26<br>1.00E-24<br>8.00E-21<br>1.00E-20<br>2.00E-18<br>7.00E-14	SymbF.scaffold3240.10<br>SymbF.scaffold690.22<br>SymbF.scaffold5147.8	2.00E-82<br>9.00E-37<br>3.00E-13
UniProt ID: B2IXF8<br>Gene unknown<br>Gene ID in *N. punctiforme*: Npun_R1259	No hits	N/A	No hits	N/A	No hits	N/A	No hits	N/A

Supplementary Table 17. Statistics of preliminary genome assemblies.

	***Symbiodinium goreaui* SCF055-01 (clade C, type C1)**			***Symbiodinium kawagutii* CS-156 (=CCMP2468, clade F)**		
	**ALLPATHS-LG**	**CLC Genomics Workbench**	**SPAdes***	**ALLPATHS-LG**	**CLC Genomics Workbench**	**SPAdes**
%G+C	45.40	45.36	44.53	45.40	45.91	45.74
Total number of scaffolds	28,243	41,412	1,906,899	6,305	85,340	501,627
Total assembled bases (bp)	1,011,466,282	1,060,349,155	1,452,039,755	913,085,015	1,106,975,017	1,300,991,596
N50 length of scaffolds (bp)	113,414	101,436	42,733	458,350	272,097	243,842
Maximum scaffold length (bp)	8,454,000	8,337,350	8,340,000	5,432,874	5,507,473	6,118,942
Total number of contigs	110,037	270,205	2,185,232	53,712	182,229	615,029
N50 length of contigs (bp)	15,777	6,805	4,252	31,411	35,785	40,446
Maximum contig length (bp)	2,989,000	3,449,000	1,859,000	2,124,640	1,933,067	1,245,445
Number of scaffolds > 50 Kb	6,075	6,206	5,830	2,694	4,264	4,100
% genome in scaffolds > 50 Kb	78.05	73.64	46.86	97.32	87.82	76.06
% gap	21.00	12.89	8.81	7.83	14.63	19.80
% CEGMA genes (eukaryote)	75.11	76.20	85.37	74.45	83.19	74.89
% BUSCO genes (eukaryote)	37.62	37.62	38.61	38.61	46.86	40.26

*: This SPAdes assembly is based on *k*-mer size 111 only, thus is more fragmented than the others.

Supplementary Table 18. Proteins used as evidence in evidence-based gene prediction.

Source	Number of sequences
RefSeq release 78 (complete)	49,301,516
Symbiodinium minutum (Shoguchi et al. 2013)	47,014
Symbiodinium kawagutii (Lin et al. 2015)	36,850
Symbiodinium microadriaticum (Aranda et al. 2016)	49,109
Symbiodinium kawagutii MMETSP0132	12,602
Symbiodinium sp. CCMP421; MMETSP1110	72,821
Symbiodinium sp. CCMP2430; MMETSP1115	44,145
Symbiodinium sp. Mp.; MMETSP1122	44,283
Symbiodinium sp. C1; MMETSP1367	43,592
Symbiodinium sp. C15; MMETSP1370	35,777
Symbiodinium sp. D1A; MMETSP1377	45,153
**Total number of sequences**	**49,732,862**

Supplementary Note 1

Nuclear genomes of *S. goreaui* and *S. kawagutii*

The G+C contents of our assembled genomes of *S. goreaui* (43.8%) and *S. kawagutii* (45.6%) are comparable to the earlier *S. kawagutii* assembly (44.0%)[1] and to *S. minutum* (43.6%)[2], and lower than for *S. microadriaticum* (50.5%)[3]. Supplementary Fig. 1A shows the extent of mapped sequence reads from *S. goreaui* and from *S. kawagutii* to each assembled *Symbiodinium* genome. Most reads (>78%) mapped to the corresponding assembly. Only about 16.5% of reads from *S. goreaui* mapped to the two *S. kawagutii* genomes, and conversely 17.6% of *S. kawagutii* reads to *S. goreaui*. This is in contrast to 4.1% of *S. goreaui* reads and 10.3% of *S. kawagutii* reads that mapped to *S. microadriaticum*. These results indicate a high extent of dissimilarity among *Symbiodinium* genomes, greatest between representatives of Clades C and A, and least (although not by far) between C and F.

We adopted a comprehensive *ab initio* approach for predicting genes, combining both evidence-based and unsupervised methods (Methods). Average gene lengths (7671 bp in *S. goreaui*, 6646 bp in *S. kawagutii*) are intermediate between the shortest (3788 bp in the earlier *S. kawagutii* genome)[1] and longest average (12,898 bp in *S. microadriaticum*)[3]. In addition, we observed similar codon usage (Supplementary Fig. 4) and amino acid profiles (Supplementary Fig. 5) among the genes of *S. goreaui, S. kawagutii* and *S. microadriaticum*; the latter shows a slight bias towards high-G+C codons. *S. minutum*[2] shows a distinctive codon usage profile vis-à-vis the others, with substantially higher contents of arginine, serine and tryptophan in predicted protein sequences (Supplementary Fig. 5).

Proportions of canonical (GC) and non-canonical 5′-donor splice sites (GA, GT) in *Symbiodinium* genomes are shown in Supplementary Table 9. These splice sites occur in similar proportion in the genomes of *S. goreaui* and *S. kawagutii*, with GA << GC < GT (e.g. 19.5% GA, 36.1% GC and 44.4% GT in *S. kawagutii*); a similar pattern was observed in *S. minutum*[2], whereas in *S. microadriaticum*[3] the canonical GC is more prominent (21.9% GA, 52.1% GC and 26.0% GT). Non-canonical 5′-donor sites were not explicitly considered in the gene-prediction process for the earlier *S. kawagutii* genome, although a dominance of GT sites (65.6%) was observed. In all *Symbiodinium* genomes, a non-canonical G usually immediately follows the acceptor splice site (Supplementary Table 9 and Supplementary Fig. 6). Thus *Symbiodinium* of Clades C and F use donor splice sites similarly to Clade B, but

different than Clade A; this is likely related to the higher G+C content in the *S. microadriaticum* genome (50.51%) than in the others (43.46-45.59%).

Plastid genomes of *S. goreaui* and *S. kawagutii*

Plastid genomes of dinoflagellates occur as minicircles each with one or more protein-coding genes, plus a non-coding region that contains a highly conserved core[4-6]. We identified putative minicircle sequences with plastid-encoded genes in each of our two genomes: nine sequences encoding 14 genes in *S. goreaui* (Supplementary Table 4), and 13 sequences encoding 13 genes in *S. kawagutii* (Supplementary Table 5). A highly conserved core region was identified, of 79 bp and 41 bp for *S. goreaui* and *S. kawagutii* respectively (Supplementary Table 7). We also identified putative "empty" minicircles (two in *S. goreaui*, one in *S. kawagutii*) that do not encode any gene. Empty minicircles have been described in other dinoflagellates, but were not seen in *Symbiodinium* type C3[4] or *S. minutum*[7]. Plastid-encoded *psbI*, previously reported in *S. minutum* but not in *Symbiodinium* type C3, was found in minicircle-like sequences in both *S. goreaui* and *S. kawagutii*; we did not find direct evidence of circularisation in these sequences, but a core region is present in the *psbI*-encoding sequence from *S. goreaui*. Our results demonstrate that "empty" minicircles and plastid-encoded *psbI* occur in *Symbiodinium*; full-length minicircle sequences in *S. goreaui* and *S. kawagutii* remain to be validated.

Specifically from the *S. goreaui* ALLPATHS-LG assembly we recovered eight scaffolds containing all 13 known plastid-encoded genes (Supplementary Table 4) described previously for *Symbiodinium* type C3, as well as a scaffold containing the *psbI* gene described previously in *S. minutum*. Some of the genes were recovered only in part, and none of the scaffolds shows clear evidence of circularisation. Three of the gene-coding scaffolds (SC1_Plastid_1, SC1_Plastid_2 and SC1_Plastid_3) displayed characteristics that have not been described before in dinoflagellate minicircles. These scaffolds are longer than minicircles observed in other dinoflagellates, and contain multiple core regions. They also encode multiple genes, some of which are fragmentary. Although minicircles with two genes have been observed in other dinoflagellates, studies in *Symbiodinium* type C3 and *S. minutum* have not recovered any such structures[4,7].

All plastid genes except *psaA*, 16S rRNA and *psaB* were recovered in *S. goreaui*. The 16S rRNA is fragmented in *Symbiodinium*; we recovered three partial fragments encoded on

SC1_Plastid_3. Only 48% (973/2022 bp) of the *psaA* gene, representing the end of the sequence, was recovered in a single fragment on SC1_Plastid_1. One full-length and one partial (12%; 128/1029 bp) copy of the *psbA* gene are present in SC1_Plastid_2. The *psaB* gene is encoded over two fragments (representing regions of 50-1319 nt and 1502-2082 nt) that together comprise 89% (1851/2082 bp) of the length of the *psaB* described in type C3. The two fragments of the *psaB* gene are encoded sequentially, separated on the scaffold by 3 bp; the length of the missing sequence between the two fragments is 182 bp. Studies in other dinoflagellates suggest that genes such as *psaA* have undergone internal deletions as a way of reducing size to fit on a minicircle[4,5], and such a process could also have impacted the *psaB* gene described in type C3. A 79-bp core (Supplementary Table 7) was identified in *S. goreaui* and recovered in all scaffolds found to encode plastid genes. SC1_Plastid_8 contains a partial core region that is encoded at the beginning of the sequence and SC1_Plastid_9 contained a full length core region with two mismatches. The core region was used to isolate SC1_Plastid_10 that had not been previously identified as being of plastid origin. It is not circular, and a comparison with the NCBI NR database found no similarity with any known genes. The identity of this scaffold as a minicircle, and whether it is an "empty" minicircle, remain to be validated.

From the *S. kawagutii* data we recovered 13 scaffolds that contain 13 of the known plastid-encoded genes (Supplementary Table 5), with only *psbE* not recovered. Five of the scaffolds (SF_Plastid_1, SF_Plastid_2, SF_Plastid_3, SF_Plastid_4 and SF_Plastid_5) are outside the 2-4 Kbp size range observed in other dinoflagellates[4]. The 16S rRNA was found as two partial fragments encoded on two scaffolds, consistent with our current understanding of the 16S rRNA gene structure in *Symbiodinium*. The *psaB*, *atpB*, *psaA*, *psbC* and *atpA* genes were recovered as multi-copy fragments encoded on the same scaffold. For example, the *atpA* gene in SF_Plastid_5 is present in two nearly complete copies, whereas in SF_Plastid_1 six partial fragments of the *psaB* gene were recovered. Gene structures of this nature have not been observed in any other dinoflagellate.

Three gene-encoding scaffolds (SF_Plastid_3, SF_Plastid_5 and SF_Plastid_12) were found to also be circular in *S. kawagutii*. SF_Plastid_3 has a 139-bp overlap (100% identity) at each end of the sequence, SF_Plastid_5 has a 1874-bp overlap (98% identity) and SF_Plastid_12 a 45-bp overlap (100% identity). A 41-bp core was identified in *S. kawagutii* and was recovered in only six of the plastid gene-encoding scaffolds. Two previously

unidentified scaffolds (SF_Plastid_14 and SF_Plastid_15) were found to contain the *S. kawagutii* core region, with SF_Plastid_14 also found to be circular. Both scaffolds were checked against the NCBI nr database and show no similarity with any known encoded genes. Circularisation of SF_Plastid_14 occurs between the two encoded core regions that are positioned exactly at the start and end of the scaffold. The positioning of the core regions along the scaffold may be an artefact of assembly and so the identity of the scaffold, potentially as an "empty" minicircle, remains to be investigated.

In *S. goreaui* and *S. kawagutii*, some scaffolds identified as being of plastid origin show traits that have not been observed in other dinoflagellates. In both *S. goreaui* and *S. kawagutii* there are scaffolds which exceed the size expected of a minicircle (i.e. 1.3-3.0 Kbp[4,7]), and encode gene structures that vary significantly from what has been observed for plastids of other dinoflagellates. Duplications within the scaffolds (encompassing genes, core regions and non-coding regions) and scaffolds composed of multiple minicircles could for instance be assembly artifacts. The data generation and assembly strategy adopted in this study was designed to recover nuclear rather than organellar genomes. These data serve as the first analysis platform for the organellar genomes of *S. goreaui* and *S. kawagutii*, and can be extended using a PCR sequencing strategy based on targeted primer design.

The G+C contents of the coding and non-coding regions for the plastid sequences in *S. goreaui* and *S. kawagutii* are summarised in Supplementary Table 6. In the plastid minicircles of *Symbiodinium* type C3, G+C content within the coding regions (36.03%) is lower than that within the non-coding regions (48.22%)[4]. The gene-coding regions identified in our *S. goreaui* data have G+C content (36.80%) close to that published for type C3 (36.03%). The G+C content of the non-coding regions (45.64%), however, is three percentage points lower than the published data (48.22%), possibly due to the increased length of identified non-coding sequences (18,051 bp in *S. goreaui* and 11,475 bp in type C3). Our *S. kawagutii* data have G+C content in their coding (35.46%) and non-coding regions (42.56%) below that observed in type C3 and in our *S. goreaui* data. For *S. kawagutii* both the length of the recovered coding and non-coding sequence are much higher than that of type C3. Many of the scaffolds encode duplicate regions of genes, increasing the total length of the coding sequence recovered beyond what is observed for type C3.

Mitochondrial genomes of *S. goreaui* and *S. kawagutii*

We identified one scaffold from *S. goreaui,* and two from *S. kawagutii*, that contain genes known to be encoded in the mitochondrial genome of dinoflagellates. For *S. goreaui*, SC1_Mitochondria_1 (length 55,144 bp) was found to contain the *cox3*, *cox1* and *cob* genes, with a fragment of the LSU rRNA between the *cox3* and *cox1* genes. For *S. kawagutii* SF_Mitochondria_1 (length 62,663 bp) the *cox3*, *cox1* and *cob* genes were all recovered, and the LSU rRNA was found between the *cox3* and *cox1* genes. SF_Mitochondria_2 (length 35,965 bp) from *S. kawagutii* contains only the *cox1* gene. This configuration of *cox3*-LSU rRNA-*cox1-cob* is well-characterised in the apicomplexa *Plasmodium falciparum*[8] and has been observed in dinoflagellates[9,10]. The size of the mitochondrial genome in dinoflagellates has not yet been determined, but previous studies recovered a genome sequence of up to 42 Kbp[9]. SC1_Mitochondria_1 from *S. goreaui* and SF_Mitochondria_1 from *S. kawagutii* both display characteristics of dinoflagellate mitochondrial genomes but are 55,144 and 62,663 bp in length respectively. Our findings suggest that the mitochondrial genome of *Symbiodinium* is considerably larger than what has been described previously at 42 Kbp[9] in dinoflagellates.

DMSP and DMS

Dimethysulphoniopropionate (DMSP) serves as an osmolyte and antioxidant for both the alga and the coral, as a nutrient for associated bacteria[11] and as a signal in maintaining the complex interactions among holobiont partners[12]. In coral-associated *Symbiodinium*[13] and other biological systems, DMSP lyase converts DMSP into dimethylsulphide (DMS). DMS, in turn, is the major input of biogenic sulphur into the marine boundary layer[14] and can contribute to sulphate aerosols that nucleate cloud condensation, thereby lowering global temperatures[15]. DMSP concentrations are positively correlated with the thermal tolerance of *Symbiodinium*, and with bleaching tolerance of the coral *Acropora millepora*[16].

Investigating the salinity-induced production of DMSP, Lyon et al.[17] identified candidate enzymes of DMSP biosynthesis in the sea-ice diatom *Fragilariopsis cylindrus*. DMS production correlates with expression of *Alma1* (encoding DMSP lyase) in the bloom-forming cryptophyte *Emiliania huxleyi*[18,19]. Here we used BLASTP to search these sequences against our *Symbiodinium* gene models. We found three putative DMSP lyase genes in *S. goreaui,* and four in *S. kawagutii* (Supplementary Data 3 and 4); the predicted proteins show significant sequence similarity ($E < 10^{-100}$ and nearly full-length alignment) to *Alma* genes in *E. huxleyi* and the Alma1 protein described in *Symbiodinium* Clade D, indicative of capacity

to degrade DMSP into DMS. Since Raina et al.[20] showed that corals can also produce DMSP, we further examined whether coral could degrade DMSP by searching for ALMA orthologs. We find that *A. digitifera* has the molecular machinery that potentially encodes Alma1 and produce DMS. Since both coral and symbiotic alga have the genetic capacity to produce DMSP and DMS, we encourage future studies to confirm whether DMS is produced by corals and explore the mechanisms that regulate the dynamics of DMS(P) production and its influence on the complex interplay among corals, *Symbiodinium* spp. and other symbiotic microbes, especially those using DMSP as a source of sulphur.

Evolution of gene families

The results of gene gain and gene loss along *Symbiodinium* lineages with respect to the outgroup *P. glacialis* are shown in Supplementary Fig. 10. Overall we observed a higher number of gene losses in the lineage leading to *S. kawagutii* (Clade F) than in that leading to *S. goreaui* (Clade C), i.e. 3122 versus 1375 genes respectively among protein sets generated at $I = 1.0$ (Supplementary Fig. 10A). More gene losses are inferred when the clustering is more granular, e.g. 6314 versus 3482 genes at $I = 2.0$ (Supplementary Fig. 10C). In comparison, the lineages leading to *S. minutum* (Clade B) and *S. microadriaticum* (Clade A) appear to have gained more genes (186 and 218 genes respectively) than those leading to *S. kawagutii* and *S. goreaui* (97 and 69 respectively) ($I = 2.0$; Supplementary Fig. 10C). The greater inferred number of gene loss in the lineages leading to Clades C and F, and of gene gain in lineages leading to Clades A and B, while interesting, remain to be validated with more-complete genome data from *Symbiodinium*.

Supplementary References

1. Lin, S. et al. The *Symbiodinium kawagutii* genome illuminates dinoflagellate gene expression and coral symbiosis. *Science* **350**, 691-694 (2015).

2. Shoguchi, E. et al. Draft assembly of the *Symbiodinium minutum* nuclear genome reveals dinoflagellate gene structure. *Curr. Biol.* **23**, 1399-1408 (2013).

3. Aranda, M. et al. Genomes of coral dinoflagellate symbionts highlight evolutionary adaptations conducive to a symbiotic lifestyle. *Sci. Rep.* **6**, 39734 (2016).

4. Barbrook, A.C., Voolstra, C.R. & Howe, C.J. The chloroplast genome of a *Symbiodinium* sp. Clade C3 isolate. *Protist* **165**, 1-13 (2014).

5. Howe, C.J., Nisbet, R.E.R. & Barbrook, A.C. The remarkable chloroplast genome of dinoflagellates. *J. Exp. Bot.* **59**, 1035-1045 (2008).

6. Zhang, Z., Green, B.R. & Cavalier-Smith, T. Single gene circles in dinoflagellate chloroplast genomes. *Nature* **400**, 155-159 (1999).

7. Mungpakdee, S. et al. Massive gene transfer and extensive RNA editing of a symbiotic dinoflagellate plastid genome. *Genome Biol. Evol.* **6**, 1408-1422 (2014).

8. Feagin, J.E. The 6-kb element of *Plasmodium falciparum* encodes mitochondrial cytochrome genes. *Mol. Biochem. Parasitol.* **52**, 145-148 (1992).

9. Jackson, C.J. et al. Broad genomic and transcriptional analysis reveals a highly derived genome in dinoflagellate mitochondria. *BMC Biol.* **5**, 41 (2007).

10. Nash, E.A., Nisbet, R.E.R., Barbrook, A.C. & Howe, C.J. Dinoflagellates: a mitochondrial genome all at sea. *Trends Genet.* **24**, 328-335 (2008).

11. Teplitski, M., Krediet, C.J., Meyer, J.L. & Ritchie, K.B. Microbial interactions on coral surfaces and within the coral holobiont. In *The Cnidaria, Past, Present and Future: The World of Medusa and Her Sisters* (eds. Goffredo, S. & Dubinsky, Z.) 331-346 (Springer International Publishing, Cham, Switzerland, 2016).

12. Raina, J.B., Dinsdale, E.A., Willis, B.L. & Bourne, D.G. Do the organic sulfur compounds DMSP and DMS drive coral microbial associations? *Trends Microbiol.* **18**, 101-108 (2010).

13. Yost, D.M. & Mitchelmore, C.L. Dimethylsulfoniopropionate (DMSP) lyase activity in different strains of the symbiotic alga *Symbiodinium microadriaticum*. *Mar. Ecol. Prog. Ser.* **386**, 61-70 (2009).

14. Liss, P.S., Hatton, A.D., Malin, G., Nightingale, P.D. & Turner, S.M. Marine sulphur emissions. *Phil. Trans. R. Soc. B* **352**, 159-169 (1997).

15. Charlson, R.J., Lovelock, J.E., Andreae, M.O. & Warren, S.G. Oceanic phytoplankton, atmospheric sulphur, cloud albedo and climate. *Nature* **326**, 655-661 (1987).

16. Jones, G.B. & King, S. Dimethylsulphoniopropionate (DMSP) as an indicator of bleaching tolerance in scleractinian corals. *J. Mar. Sci. Eng.* **3**, 444-465 (2015).

17. Lyon, B.R., Lee, P.A., Bennett, J.M., DiTullio, G.R. & Janech, M.G. Proteomic analysis of a sea-ice diatom: salinity acclimation provides new insight into the dimethylsulfoniopropionate production pathway. *Plant Physiol.* **157**, 1926-1941 (2011).

18. Alcolombri, U. et al. Identification of the algal dimethyl sulfide-releasing enzyme: a missing link in the marine sulfur cycle. *Science* **348**, 1466-1469 (2015).

19. Alcolombri, U., Lei, L., Meltzer, D., Vardi, A. & Tawfik, D.S. Assigning the algal source of dimethylsulfide using a selective lyase inhibitor. *ACS Chem. Biol.* **12**, 41-46 (2017).

20. Raina, J.B. et al. DMSP biosynthesis by an animal and its role in coral thermal stress response. *Nature* **502**, 677-680 (2013).
