# Supplementary material for: Symbiodinium genomes reveal adaptive evolution of functions related to coral-dinoflagellate symbiosis
Source: Commun Biol. 2018 Jul 17;1:95. doi: 10.1038/s42003-018-0098-3 (PMC6123633; doi:10.1038/s42003-018-0098-3)
Supplement: Supplementary file 2 — Description of Additional Supplementary Files [file 42003_2018_98_MOESM2_ESM.pdf]

Description of Additional Supplementary Files

File name: SupplementaryData_1.xlsx

Description: **Supplementary Data 1. Recovery of CEGMA genes based on analysis of predicted proteins and genome sequences of *Symbiodinium*.**

File name: SupplementaryData_2.xlsx

Description: **Supplementary Data 2. Syntenic collinear blocks within each of the four genomes of *S. goreaui* (Sgoreaui), *S. kawagutii* (Skawagutii), *S. microadriaticum* (Smic) and *S. minutum* (Smin).**

File name: SupplementaryData_3.xlsx

Description: **Supplementary Data 3. Functional annotation of *S. goreaui* gene models.**

File name: SupplementaryData_4.xlsx

Description: **Supplementary Data 4. Functional annotation of *S. kawagutii* gene models.**

File name: SupplementaryData_5.xlsx

Description: **Supplementary Data 5. Comparison of *Symbiodinium*-specific proteins against all proteins from the 31-taxon set.**

File name: SupplementaryData_6.xlsx

Description: **Supplementary Data 6. Comparison of *Symbiodinium*-specific proteins against all proteins from the 15-taxon set.**

File name: SupplementaryData_7.xlsx

Description: **Supplementary Data 7. Positively selected *Symbiodinium* genes against the 15 taxa (14 dinoflagellates and *Perkinsus* as outgroup).**

File name: SupplementaryData_8.xlsx

Description: **Supplementary Data 8. Enriched GO terms in positively selected genes against all four *Symbiodinium* genomes.**

File name: SupplementaryData_9.xlsx

Description: **Supplementary Data 9. Putative meiosis-related genes in *Symbiodinium* genomes.**

File name: SupplementaryData_10.xlsx

Description: **Supplementary Data 10. Statistics of multiple sequence alignments used in the 15-taxon analysis for positive selection.**
